# Tuning photochromism in multivariate zinc dipyridyl-naphthalenediimide coordination polymers

**DOI:** 10.1039/d6dt02007k

**Published:** 2026-09-11

**Authors:** Adnan Ishaq, Louise Male, Neil R. Champness

**Affiliations:** a School of Chemistry, University of Birmingham Edgbaston Birmingham B15 2TT UK n.champness@bham.ac.uk

## Abstract

Multivariate compositions of zinc halide dipyridyl-naphthalenediimide coordination polymers are shown to directly tune photochromic response through modification of halide lone pair-NDI LUMO overlap.

## Abstract 2

Multivariate compositions of zinc halide dipyridyl-naphthalenediimide coordination polymers are shown to directly tune photochromic response.

## Graphical abstract

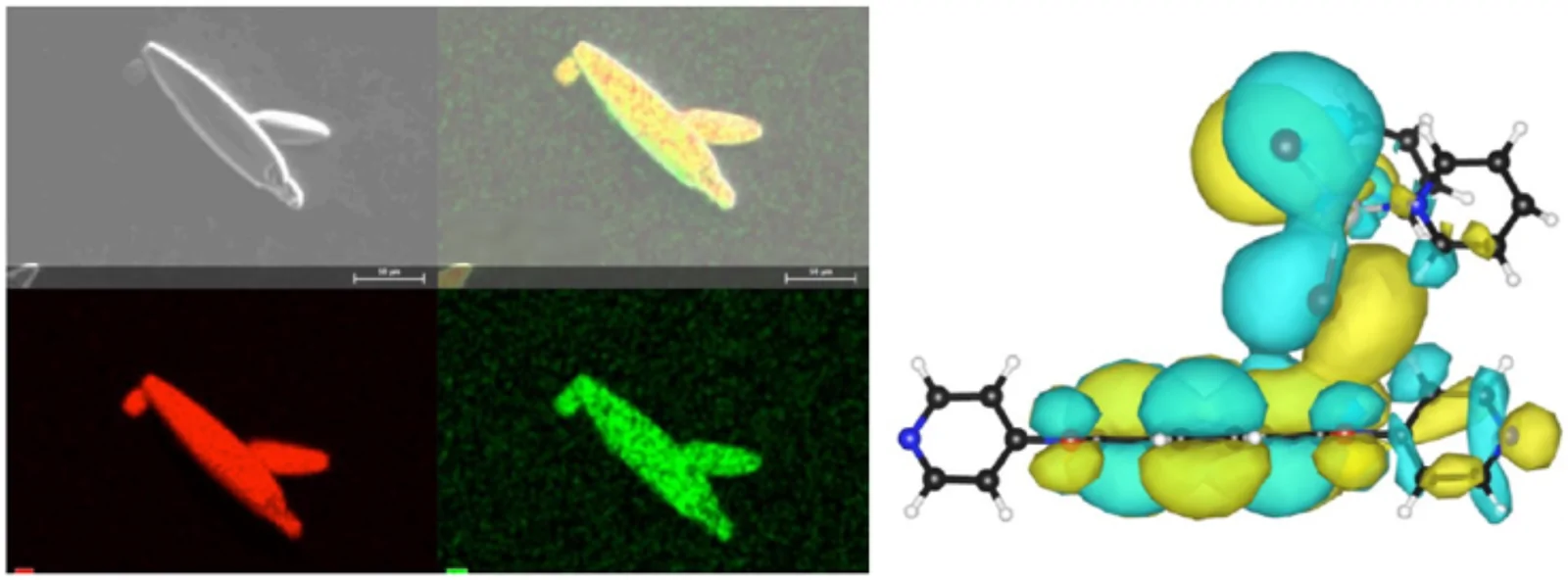

Photochromic materials have attracted widespread attention for applications in data storage, sensing and catalysis among many others.^1–9^ Naphthalenediimides (NDIs) and their longer analogues, perylenediimides, are an extensively studied family of often photochromic compounds.^10–18^ This property arises from the NDI's electron-poor aromatic core and a low-lying LUMO which makes them readily reduced to a radical anion (NDI˙^−^), a process which is usually accompanied by a change in colour of the NDI-containing material.^10–13^ In addition, NDIs are readily modified through synthetically facile substitution of functional motifs in their imide positions,^14^ or directly onto the naphthyl core.^15^ These qualities make NDIs a popular choice in constructing functional crystalline materials such as metal–organic frameworks (MOFs), hydrogen-bonded organic frameworks (HOFs) and simple co-crystals.^10,13,16–19^

Crystalline NDI containing materials are attractive as the crystal engineering approach can be employed to tune electron-donor to electron-acceptor interfaces, while crystallography can inform the researcher on the precise inter- or intra-molecular interactions which may give rise to the formation of the aforementioned NDI radical species, something which can be more challenging in the solution phase.^20–22^ In addition, crystal packing (particularly through π-stacking) can stabilise organic radicals, something which is attractive both for the investigation of radical species, but also in applications.^23,24^ Notably for crystalline materials such as coordination polymers (CPs) or MOFs, a multivariate-MOF (MTV-MOF) or multivariate-CP (MTV-CP) approach can be exploited wherein the composition of different metals or ligands can be systematically varied within a single polymer to tune the properties of the material.^25–27^

NDIs can be functionalised at the imide positions with Lewis basic group such as carboxylates or pyridyls, in turn, allowing for the construction of MOFs or CPs.^28,29^ Dipyridyl NDIs are widely studied in the synthesis of CPs with strong pyridyl binding to soft Lewis acidic metals such as Cu(ii) or Zn(ii).^30–34^ The presence of charge-balancing anions (or any electron rich species) can be an interesting component of a coordination polymer material as such species can act as inter- or intra-molecular electron donors to the NDI yielding a photochromic response.^35,36^

In this paper we present a family of isostructural, photochromic, Zn di-4-pyridyl-NDI (D4PNDI) halide (Cl^−^ or Br^−^) coordination polymers [Zn(D4PNDI)Cl_2_]_*n*_ and [Zn(D4PNDI)Br_2_]_*n*_ and multivariate analogues. By exploiting the isostructural nature of the chloride and bromide CPs, we were able to synthesise multivariate mixed-halide compositions across the Cl/Br range. We found that the magnitude of the photochromic response of these CPs scales with bromide content. A clear composition-property relationship is established and demonstrates an approach for tuning functional properties in MTV-CPs. Our observations are supported by DFT calculations which show lone pair overlap with the NDI π* LUMO is more effective for the Br^−^ anion, facilitating more efficient electron transfer.

High quality single crystals of the desired CPs, [Zn(D4PNDI)X_2_]_*n*_ (X = Cl, Br), were crystallised from dimethylacetamide (DMAc) solutions of Zn halides (Cl, Br or mixtures thereof) and dipyridyl-NDI ligand (D4PNDI)^24^ at room temperature over the course of 1 week. Single-crystal X-ray diffraction was used to determine the crystal structures of the fully Cl, fully Br and mixed halide coordination polymer crystals. The crystal structure and photochromic behaviour of the chloride analogue, [Zn(D4PNDI)Cl_2_]_*n*_, has been reported previously^31^ but is discussed herein for the purposes of comparison. It was found that [Zn(D4PNDI)Cl_2_]_*n*_, [Zn(D4PNDI)Br_2_]_*n*_ and [Zn(D4PNDI)Cl_0.4_Br_1.6_]_*n*_ are all isostructural and crystallise in the space group *P*2/*c*. The asymmetric unit contains half the D4PNDI ligand and one coordinated halide as well as a DMAc molecule. Interestingly, the unit cell parameters of the mixed-halide example are intermediate to the mono-halide examples.

The D4PNDI ligand bridges two tetrahedral ZnX_2_ nodes producing a 1D coordination polymer (Fig. 1) with a distinct zig-zag arrangement similar to that observed by Fu *et al.*^36,37^ The zig-zag 1D coordination polymers interdigitate leaving the disordered DMAc molecule within a small cavity. Notably, the halides form anion–π interactions with the NDI imide ring, on an adjacent polymer, at a separation of 3.4706(19) Å and 3.494(2) Å for the Cl and Br structures respectively (X^−^⋯N; Zn–X⋯N = 154.45(4)° (Cl), 155.44(4)° (Br)), providing a route for anion to NDI electron transfer. No π-stacking is observed between NDI units.

**Fig. 1:**
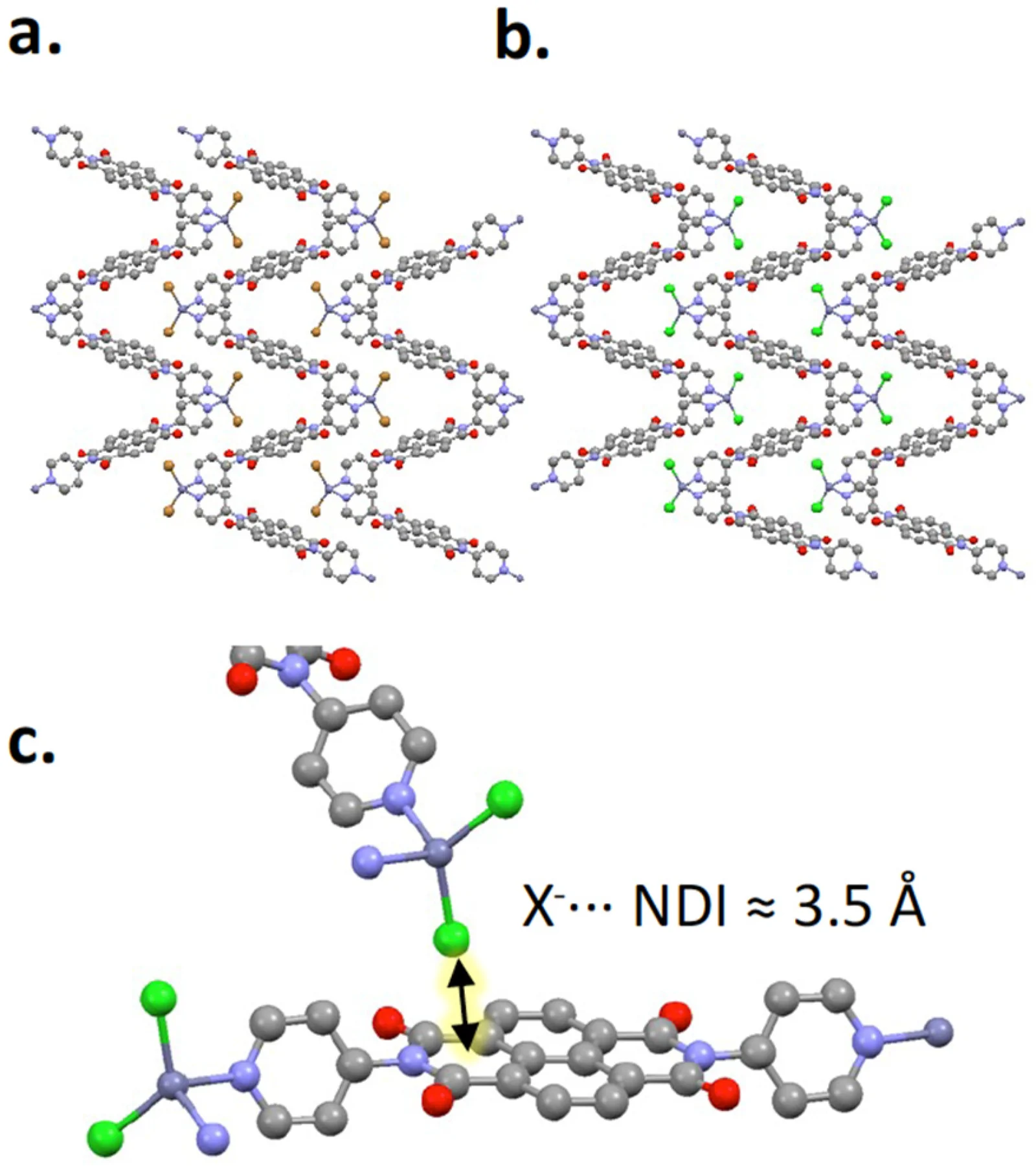
(a) crystal packing of [Zn(D4PNDI)Br_2_]_*n*_, (b) crystal packing of [Zn(D4PNDI)Cl_2_]_*n*_, (c) short X^−^⋯NDI contact.

These materials were observed to be photochromic upon irradiation by a 365 nm UV lamp, undergoing a colour change from beige to reddish-brown. We observed that [Zn(D4PNDI)Br_2_]_*n*_ underwent a larger colour change than [Zn(D4PNDI)Cl_2_]_*n*_ under the same excitation conditions, suggesting that the less electronegative, more polarisable bromide anion acts as a better electron donor than the chloride analogue due to the combination of higher energy donor orbitals and greater donor–acceptor orbital overlap. An observation which is also made by Fu *et al.* in related systems.^37^ Additionally, this confirms that the halide acts as the electron donor rather than guest DMAc molecules.

Using UV-vis spectroscopy, the magnitude of the photoexcitation of the two coordination polymers with just one type of halide, Cl^−^ or Br^−^, were investigated. Analysis of the UV-vis spectra upon irradiation show the growth of a broad underlying absorption, however a distinct band at 606 nm (Fig. 2) is observed which can be attributed to the formation of the NDI radical anion based on literature precedent.^9,38–40^ The decay kinetics were investigated after irradiation for 120 s. By plotting the normalised (between the excited and ground states) absorbance at 606 nm we can see that the bromide CP, [Zn(D4PNDI)Br_2_]_*n*_, has a significantly longer half-life compared to the Cl^−^ analogue (approximately 1960 s compared to 640 s) (Fig. S7 and S8). This difference is attributed to the greater electronegativity of the Cl^−^ with back-electron transfer from the NDI˙^−^ radical to the halide occurring more quickly, depleting the population of the radical over a shorter timeframe.

**Fig. 2:**
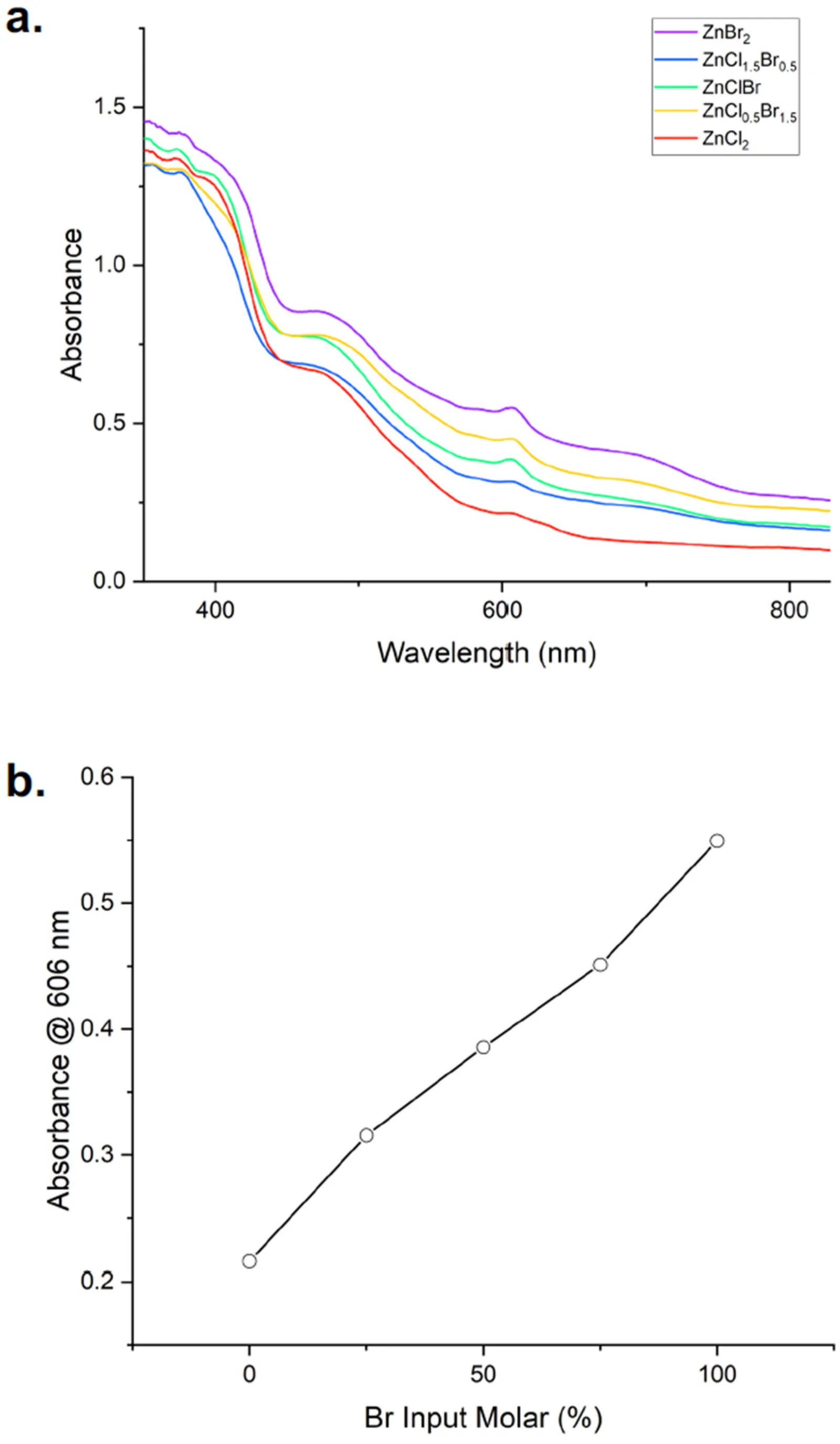
The increase in magnitude of photochromic response upon irradiation by a 365 nm UV lamp with increase in Br^−^ input feed ratio. (a) UV-vis spectra following irradiation showing growth of a band at 606 nm, (b) plot demonstrating the increased absorbance at 606 nm with increasing CP bromide content.

Having established that varying the halide component affected the photochromic behaviour, we investigated the effect of bromide content on the photochromic response in MTV-CPs. Due to the isostructural crystal structures of the two CPs, we produced MTV-CPs with mixed halide content. Samples were prepared using mixtures of ZnBr_2_ and ZnCl_2_ in the reaction, with proportions of 25 : 75%, 50 : 50% or 75 : 25% ZnBr_2_ : ZnCl_2_.

Energy dispersive X-ray spectroscopy (EDX) was employed to determine the relative halide compositions at the different molar input ratios (Fig. 3). We found that the samples, prepared under the crystallisation conditions employed consistently across batches, favoured Br^−^ inclusion. For example, when the proportion of Br^−^ used was 25%, the crystals were found to contain approximately 52% bromide (Fig. 3b). The relationship illustrated in Fig. 3b provides a guide for the preparation of samples with specific Cl^−^/Br^−^ ratios shown. This may be explained by the stronger anion–π interactions between bromide and the neighbouring NDIs, as discussed elsewhere herein. Potential surface effects were excluded by collecting EDX spectra on crushed samples and all quantification was conducted using a P/B-ZAF model to account for the greater atomic number, absorption and fluorescence effects that would be expected from Br compared to Cl. Additionally, EDX maps were collected on whole crystals which confirm homogenous mixing of the halides within the crystal, at the resolution of the experiment. Powder X-ray diffraction confirms that the samples are isostructural despite their differing composition, and that they are phase pure (Fig. 4).

**Fig. 3:**
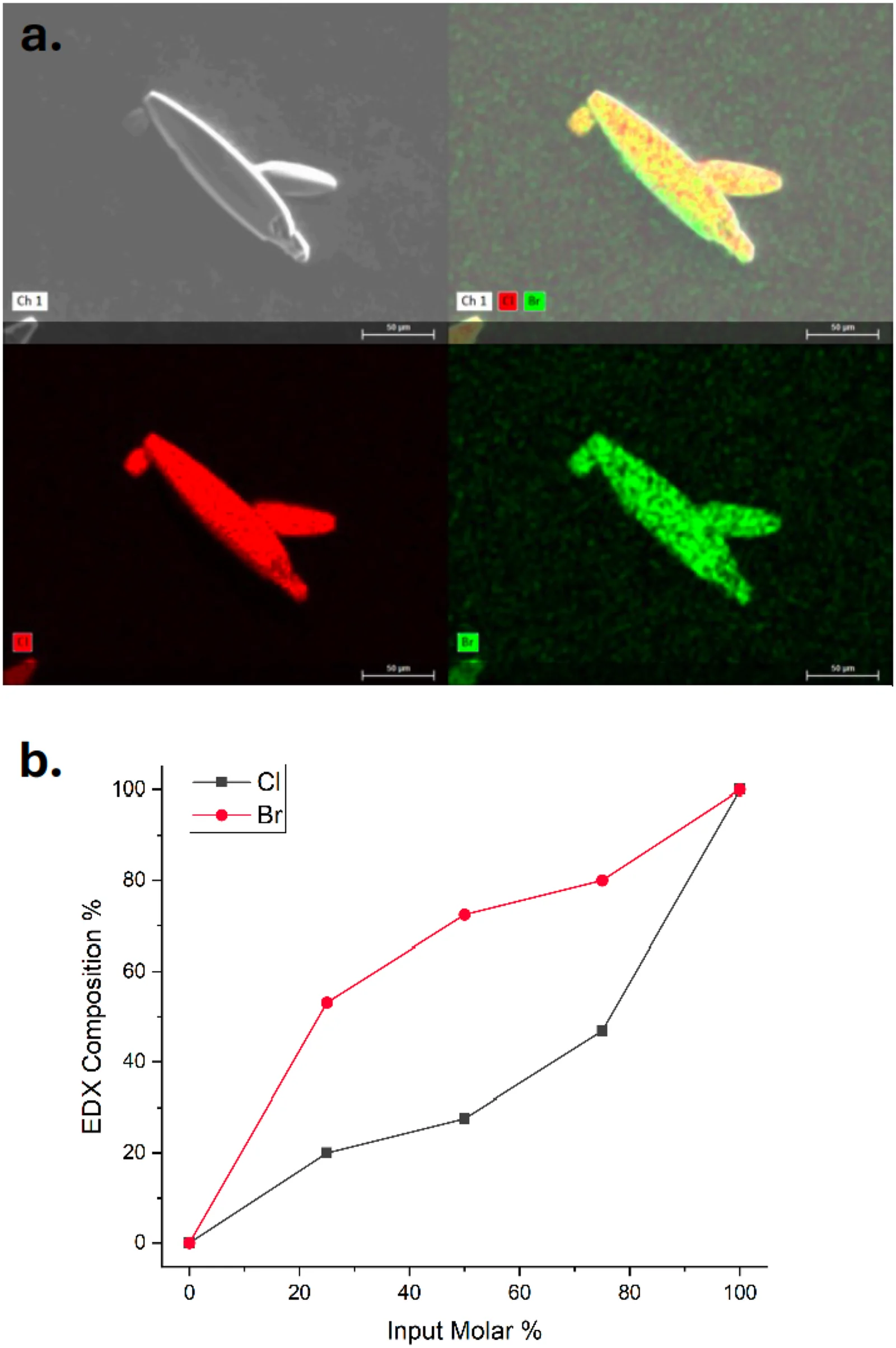
(a) EDX map of a single crystal of [Zn(D4PNDI)Cl_0.55_Br_1.45_]_*n*_ shows homogenous distribution of halide anions, (b) the effect of varying the input molar % on the composition of the coordination polymer crystals.

**Fig. 4:**
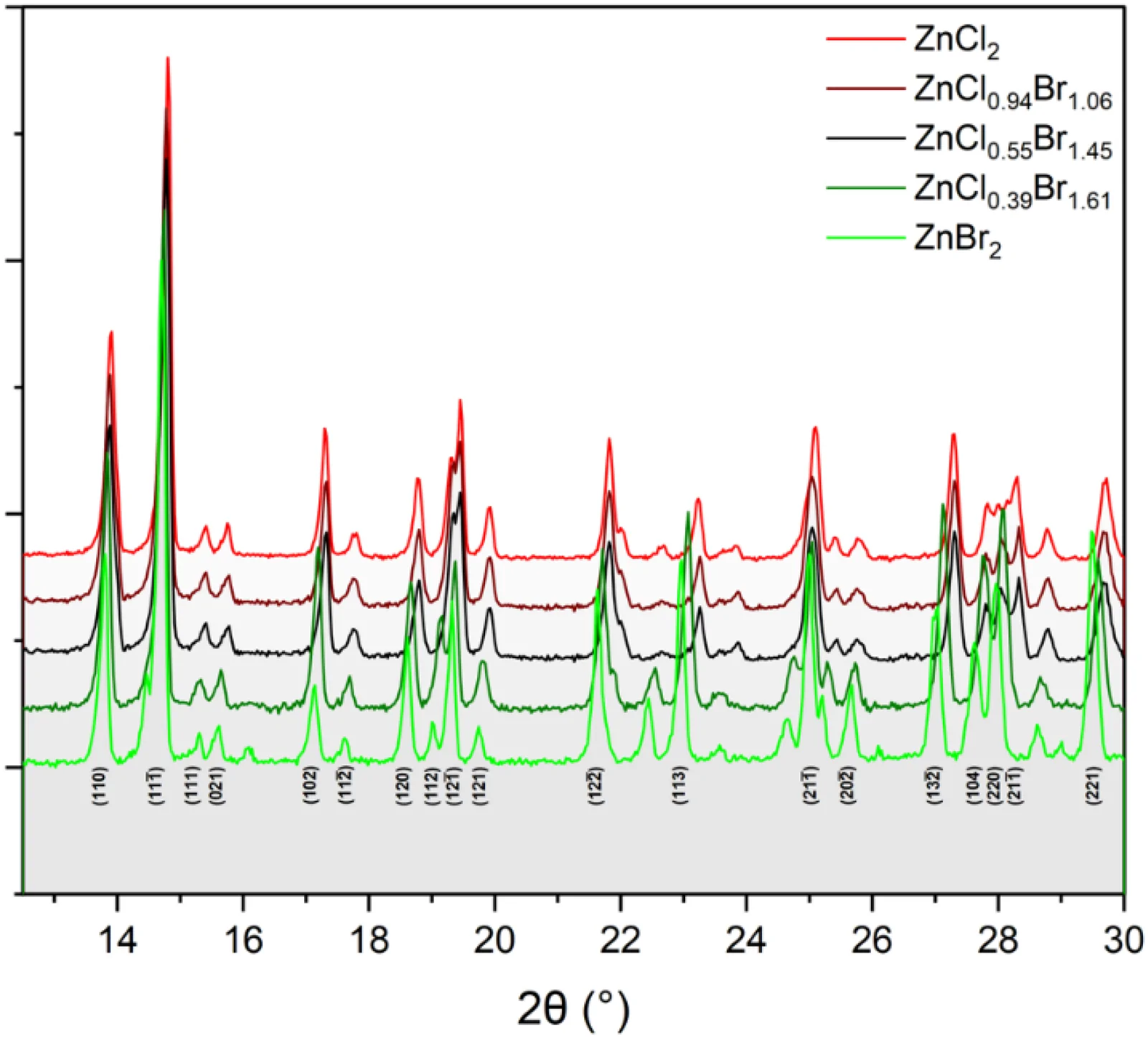
PXRD demonstrates the isostructural nature of the multivariate coordination polymers.

Variation in the Cl/Br composition in the MTV-CPs leads to concomitant variation in photochromic responses, see Fig. 2a. We observe that the photochromic response increases with increasing bromide content, with enhanced intensity of the absorption observed at 606 nm, both when measured relative to the input molar ratio, and the halide composition measured by EDX. This result demonstrates that relative halide composition within isostructural CPs is a handle with which one can tune the emergent properties of the material.^41^

In order to understand the enhanced photochromic behaviour with increasing Br^−^ content, DFT calculations were employed, at the B3LYP D3BJ/def2-SVP level of theory, to calculate the molecular orbitals (MO) in simple cluster models (Fig. 5) derived from the SCXRD structure solutions.^42–44^ The clusters employed comprise of a D4PNDI molecule which interacts with a discrete dipyridyl dihalide zinc(ii) complex in the same fashion as seen in the coordination polymers. The electron donor was simplified to a dipyridyl halide complex for ease of calculation, however this would not be expected to affect the results significantly. We found that for both the ZnCl_2_ and ZnBr_2_ clusters, the NDI LUMOs were effectively degenerate, while the 3 highest energy HOMOs were consistently higher in energy for the bromide analogue. This distinct difference in HOMO energy levels between bromide and chloride analogues confirms that the energy gap for electron transfer from the halide lone pair, to the NDI LUMO is minimised for bromide compared to chloride, leading to more efficient electron transfer.^45^ Energy gaps between halide LP HOMO and NDI π* LUMO were calculated to be 3.02 and 2.82 eV for the ZnCl_2_ and ZnBr_2_ moieties respectively, lower than the energy of the photoexcitation wavelength of 365 nm (3.40 eV). Plotting the isosurfaces of the three high lone pair character MOs (Fig. 5) provides visual and qualitative confirmation that the Br-NDI π* overlap is greater than for Cl-NDI π*, which we attribute to the greater polarizability of the bromide anions, in addition to effective donor–acceptor orbital overlap.

**Fig. 5:**
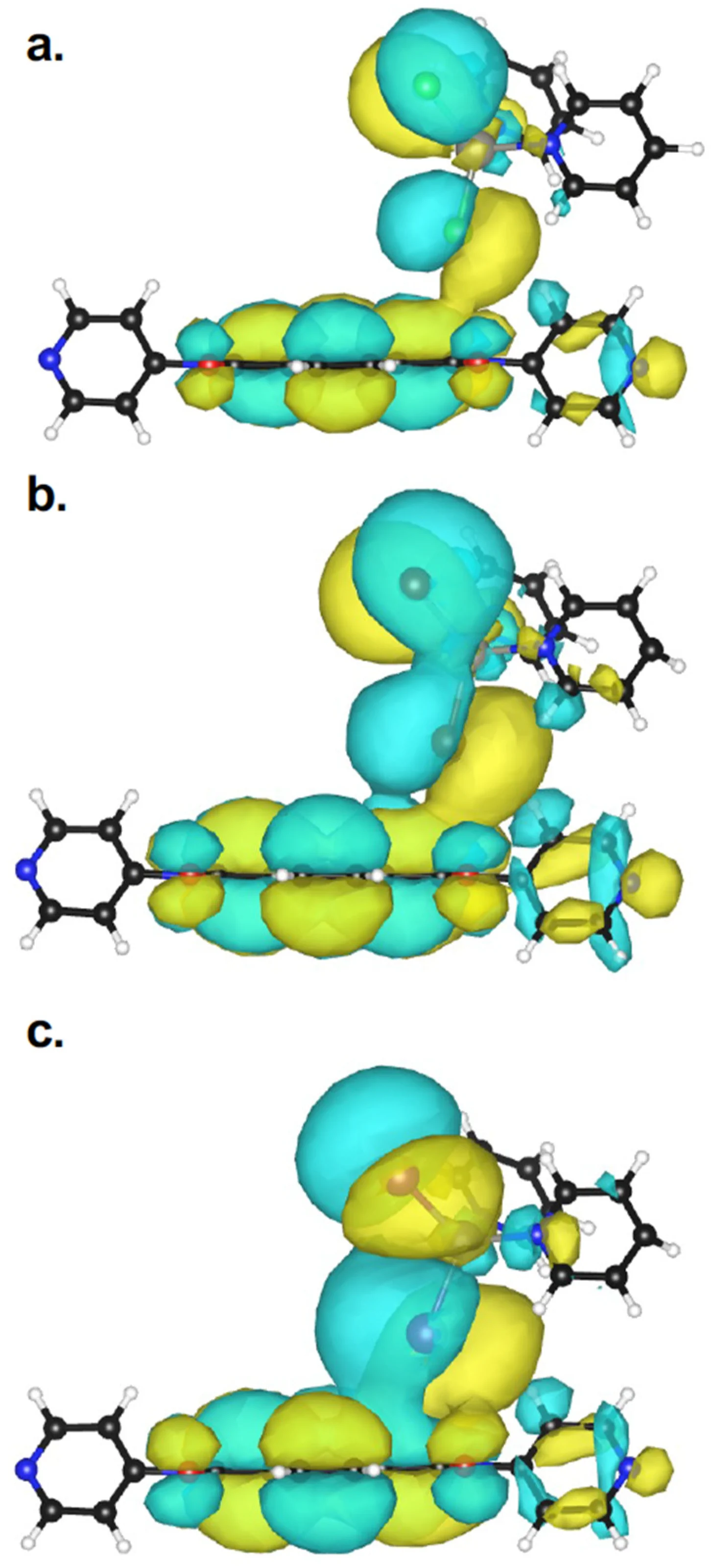
Isosurfaces showing halide lone pair and NDI π* orbital models (a) [Zn(D4PNDI)Cl_2_]_*n*_; (b) [Zn(D4PNDI)Br_2_]_*n*_ and (c) a hypothetical model of [Zn(D4PNDI)I_2_]_*n*_. Models show greater overlap for the bromide, rather than chloride, plotted as isosurfaces with the NDI LUMO at a value of 0.01.

Attempts to synthesise an analogous ZnI_2_ structure were unsuccessful, but by employing a solvent-layering crystallisation method we were able to isolate yellow block crystals of a [Zn(D4PNDI)I_2_]_*n*_ coordination polymer (Fig. S6) (in poor yield) similar in structure to that reported by Dai *et al.*^10^ but with DMAc contained within the channels rather than *N*-methyl pyrrolidone (NMP), and slightly different unit cell parameters. Unsurprisingly, in line with the findings of Dai *et al.*, we also found this compound to be non-photochromic which raises the question why iodide appears to subdue photochromism in a number of similar systems.^10,46–48^

To approach this question, DFT was employed. First, we conducted calculations on a hypothetical zinc iodide compound based on the chloride and bromide CPs reported herein, where Cl/Br are replaced by I, then the Zn–I bond lengths were optimised. As expected, the energy of the iodide LP orbitals (Fig. 5) was significantly higher, yielding a much smaller energy gap between donor and acceptor units which would be expected to yield a photochromic material. In addition, we found that the energy of the NDI LUMO was effectively degenerate with the Cl and Br compounds.

The hindering effect of iodide on the photochromic response in these materials has been previously rationalised by a reduction in π-acidity of the NDI due to electron density donation from the iodide.^10^ The DFT results are at odds with this explanation, with the NDI's LUMO energy effectively unchanged. Thus we conclude that the absence of photochromism in these iodide systems is due to a heavy atom effect promoting non-radiative decay pathways within the system, as previously proposed by other studies.^46–48^

In conclusion, we have synthesised a series of multivariate pyridyl-NDI ZnX_2_ CPs, where X = Cl, Br or variable compositions of these halides. These materials demonstrate reversible photochromic behaviour through anion-NDI electron transfer to form NDI radical anions. We observed that the bromide containing example had a greater response under the same excitation conditions compared to the chloride. Iodide containing analogues were found to be non-photochromic under the same conditions. Given the isostructural nature of [Zn(D4PNDI)Cl_2_]_*n*_ and [Zn(D4PNDI)Br_2_]_*n*_, we were able to synthesise multivariate halide materials and demonstrated that this is an approach to tuning the magnitude of the photochromic response in the resultant material. EDX measurements of the various compositions provide a composition plot which may be used in future experiments to produce a coordination polymer of a targeted halide composition, and ultimately photochromic behaviour. Finally, rationale is provided in the form of DFT calculations which demonstrate that the MO energy levels are higher for the bromide analogue, with a higher degree of orbital overlap. As such, we have demonstrated a strategy for the tuning of related materials by variation of their halide content.

## Conflicts of interest

There are no conflicts to declare.

## Supplementary Material

DT-055-D6DT02007K-s001

DT-055-D6DT02007K-s002

DT-055-D6DT02007K-s003

DT-055-D6DT02007K-s004

DT-055-D6DT02007K-s005

DT-055-D6DT02007K-s006

## Data Availability

Supplementary information (SI): details of synthesis and crystal growth, details of crystallographic measurements and other characterisation techniques. See DOI: https://doi.org/10.1039/d6dt02007k. CCDC 2560041, 2560042, 2560072 and 2579247 contain the supplementary crystallographic data for this paper.^49*a*–*d*^
