# Supplementary material for: Tuning photochromism in multivariate zinc dipyridyl-naphthalenediimide coordination polymers
Source: Dalton Trans. 2026 Sep 11;55(38):13931–5. doi: 10.1039/d6dt02007k (PMC13592237; doi:10.1039/d6dt02007k)
Supplement: Supplementary file 1 [file DT-055-D6DT02007K-s001.pdf]

Supplementary Information (SI) for Dalton Transactions.
This journal is © The Royal Society of Chemistry 2026

Supporting Information for:

Tuning Photochromism in Multivariate Zinc Dipyridyl-Naphthalenediimide Coordination Polymers

Adnan Ishaq, Louise Male and Neil Champness

Synthesis	Page 2
Single crystal X-ray Diffraction and additional figures	Page 3
Diffuse reflectance UV-Vis spectroscopy	Page 12
Energy Dispersive X-ray Spectroscopy (EDX)	Page 14
Computational Modelling	Page 15
Powder X-ray Diffraction (PXRD) Studies	Page 17
Thermogravimetric Analysis (TGA)	Page 18
Solid-state FTIR spectroscopy	Page 18
References	Page 19

Experimental

Materials

4-aminopyridine, 1,4,5,8-Naphthalenetetracarboxylic dianhydride (NTCDA), zinc chloride and zinc bromide were all purchased from either Sigma Aldrich, Alpha Aesar or Fluorochem and were used without further purification.

Synthesis of 4-pyridyl naphthalene diimide (D4PNDI)

Synthesis of the pyridyl NDI ligand followed a slightly modified literature procedure.S1 To a 50 mL round-bottom flask containing 20 mL DMF, the amine component (7.5 mmol, 700 mg) was added and this was heated to 100 °C with stirring until completely dissolved giving rise to an orange-brown solution. To this solution NTCDA (3 mmol, 804 mg) was added and heated to 140 °C. The solution turns from a beige suspension to a brown solution as the products react. After 12 hours, the reaction was allowed to cool very slowly to room temperature yielding red/orange crystals. The solid component was then collected by vacuum filtration and washed with cold DMF (3x 5 mL), acetone (3 x 5 mL) and diethyl ether (3 x 5 mL). The product was then collected and dried for 24 hours in a vacuum desiccator before use.

69 % yield, red/orange crystals, ^{1}H NMR (300 MHz, DMSO-d_6, D_2SO_4) 9.34 – 9.18 (m, 2H), 9.26 (d, J = 6.6 Hz, 4H), 8.81 (s, 4H), 8.34 (d, J = 7.2 Hz, 4H).

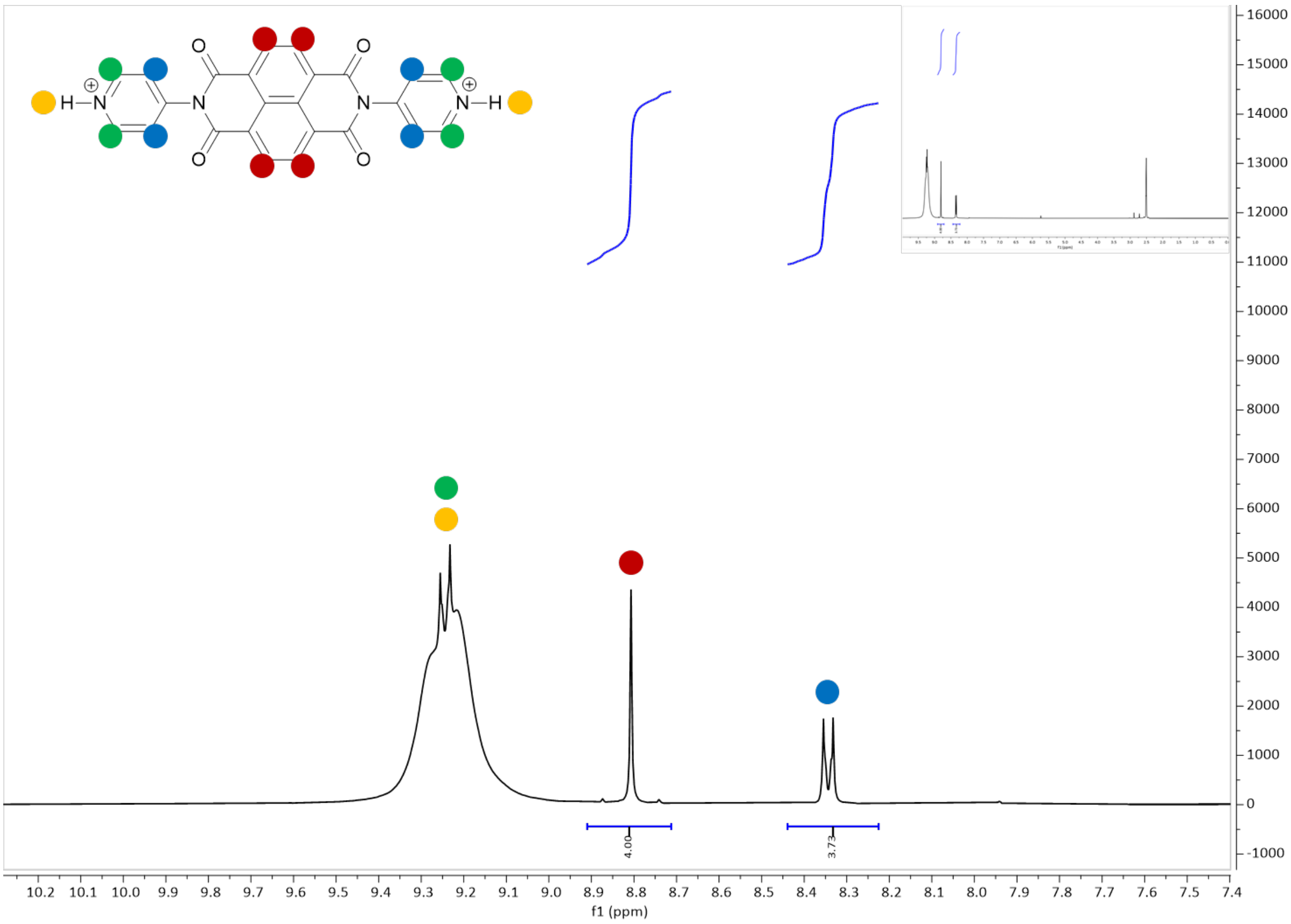

Figure S1. *^{1}H NMR spectrum of [D4PNDIH$_2$]SO_4 in DMSO-d_6/D_2SO_4.*

Crystallisation

Crystals of $[Zn(D4PNDI)X_2]_n$ were obtained by dissolving 0.125 mmol (5eq) of $ZnCl_2$ or $ZnBr_2$ (or ratios thereof) in 1.5 mL dimethylacetamide (DMAc). This was fully dissolved by sonication before 0.025 mmol (10.7 mg, 1eq) of the pyridyl naphthalene diimide ligand was added, as this dissolves it yields an orange solution. This solution is then placed aside for a week in an uncapped vial, after which, crystals of the coordination polymer are present in the vial.

A large excess of the metal is used to buffer against a solution/crystal depletion effect. This is particularly important for the multivariate examples. Furthermore, we found that an excess of the zinc halide aids in the dissolution of the NDI component.

Crystals of $[Zn(D4PNDI)I_2]_n$ were obtained by dissolving ZnI_2 (10 mg, 0.031 mmol) in 0.5 mL methanol. D4PNDI (4.2 mg, 0.01 mmol) was dissolved in 1 mL of dry DMAc as it was found that this compound does not dissolve unless the solvent is suitably dry. The DMAc ligand solution was placed in the bottom of an NMR tube followed by 0.5 mL of a 50:50 MeOH/DMAc buffer layer. On top of the buffer layer, the MeOH ZnI_2 was layered carefully. The NMR tube was then placed in a dark cupboard and after 2 weeks yellow block crystals of $[Zn(D4PNDI)I_2]_n$ had formed in very low yield.

Single Crystal X-ray Diffraction

The datasets for all 4 crystal structures were measured on a Rigaku XtaLAB Synergy diffractometer using a HyPix detector. This data collection was driven and processed and an absorption correction was applied using CrysAlisPro. Using OLEX2,S5 the structures were solved using ShelXT,S6 and were refined by a full-matrix least-squares procedure on F2 in ShelXL.S7 All non-hydrogen atoms were refined with anisotropic displacement parameters.

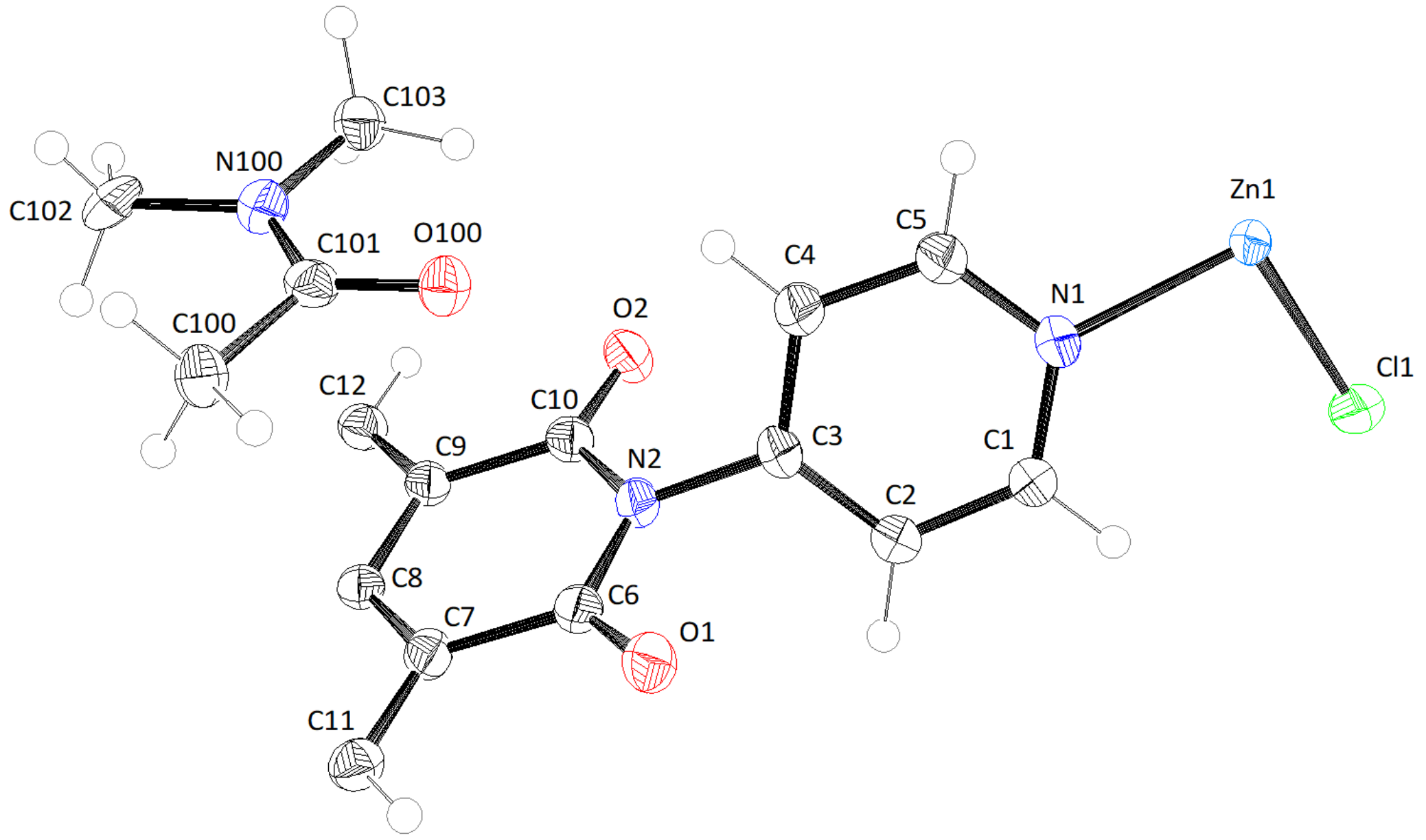

Figure S2. *ORTEP plot of asymmetric unit of [Zn(D4PNDI)Cl$_2$]$_n$. Ellipsoid probability is set at 50%.*

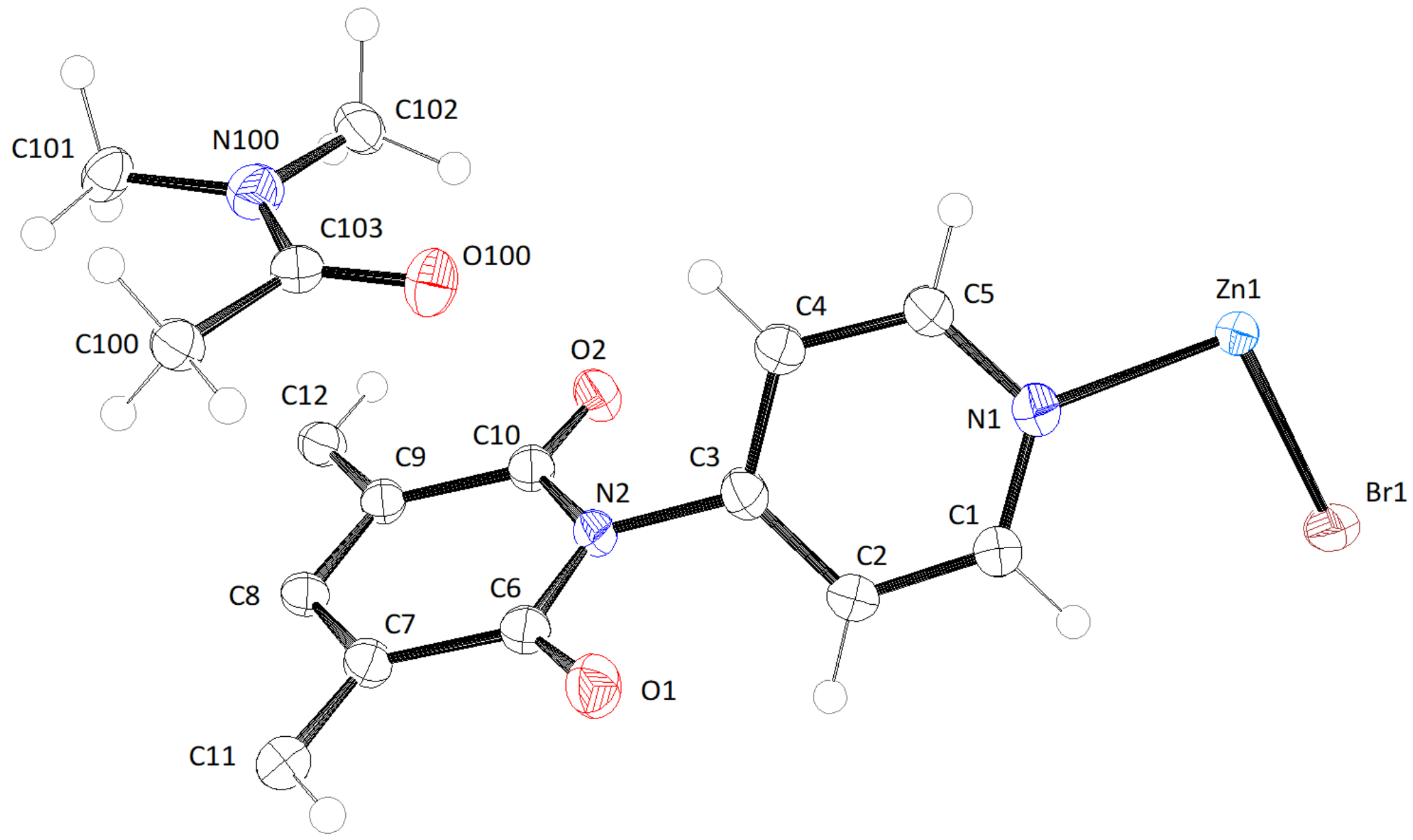

Figure S3. *ORTEP plot of asymmetric unit of [Zn(D4PNDI)Br$_2$]$_n$. Ellipsoid probability is set at 50%.*

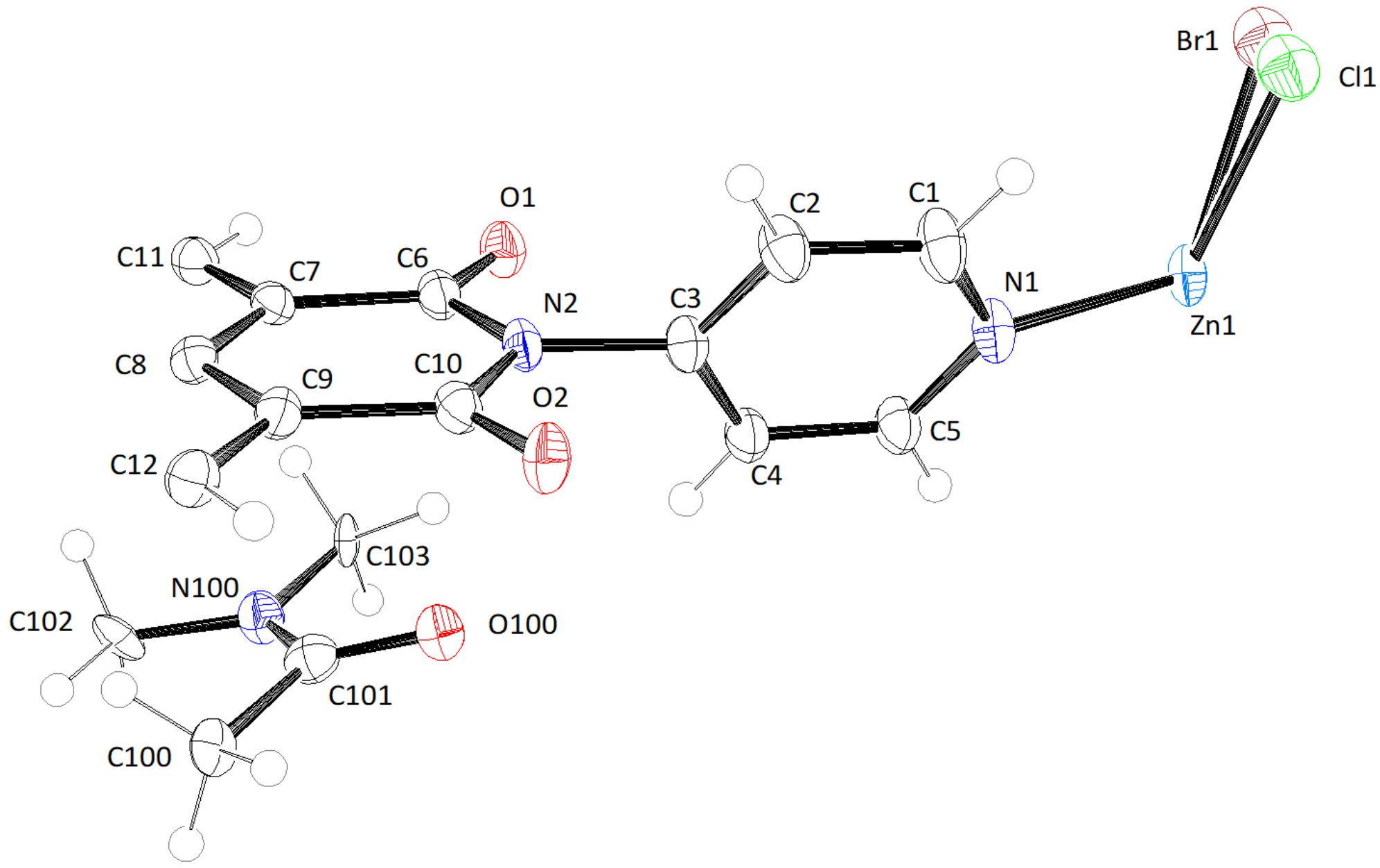

Figure S4. *ORTEP plot of asymmetric unit of [Zn(D4PNDI)Cl$_{0.4}$Br$_{1.6}$]$_n$. Ellipsoid probability is set at 50%.*

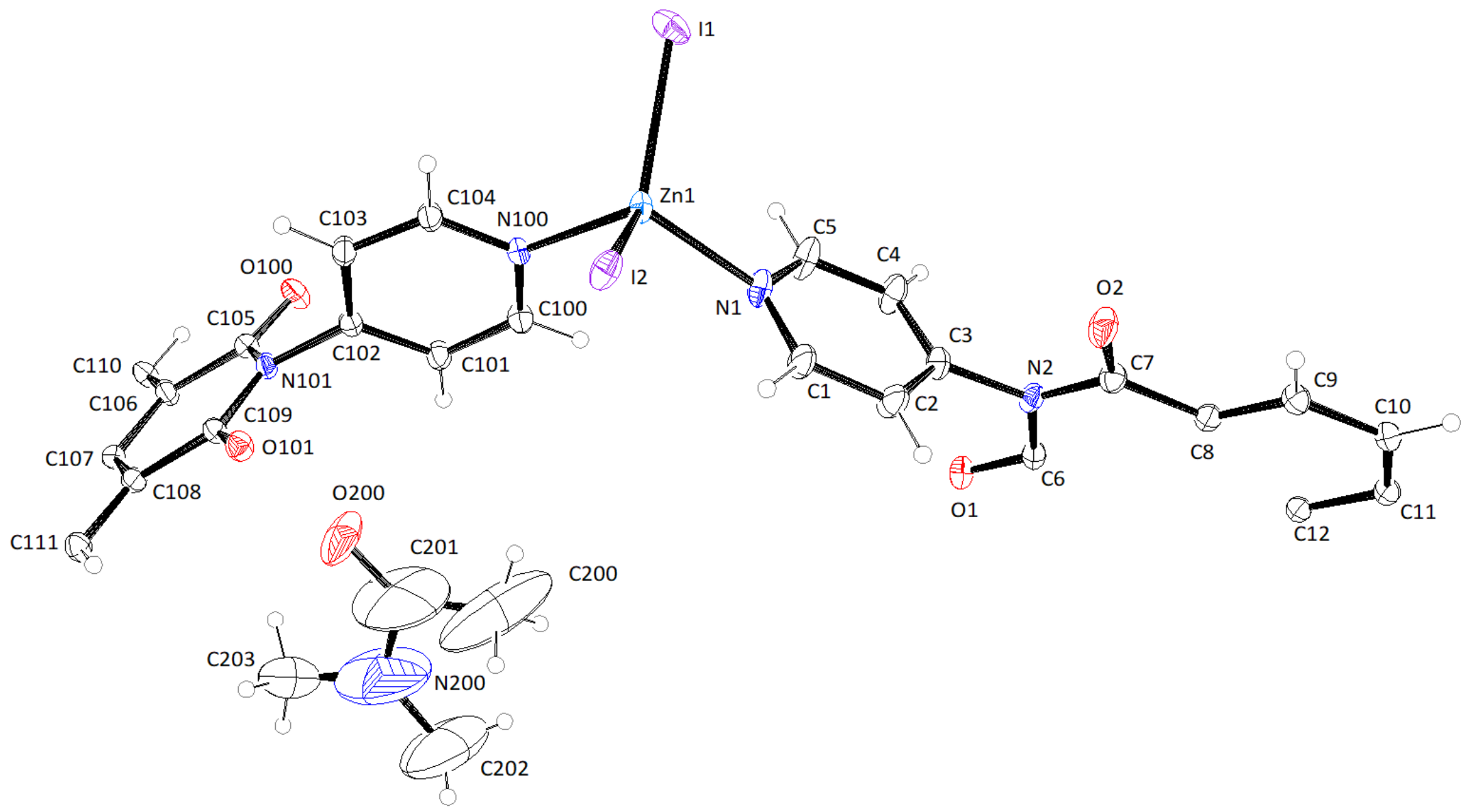

Figure S5. *ORTEP plot of asymmetric unit of [Zn(D4PNDI)I$_2$]$_n$. Ellipsoid probability is set at 30%.*

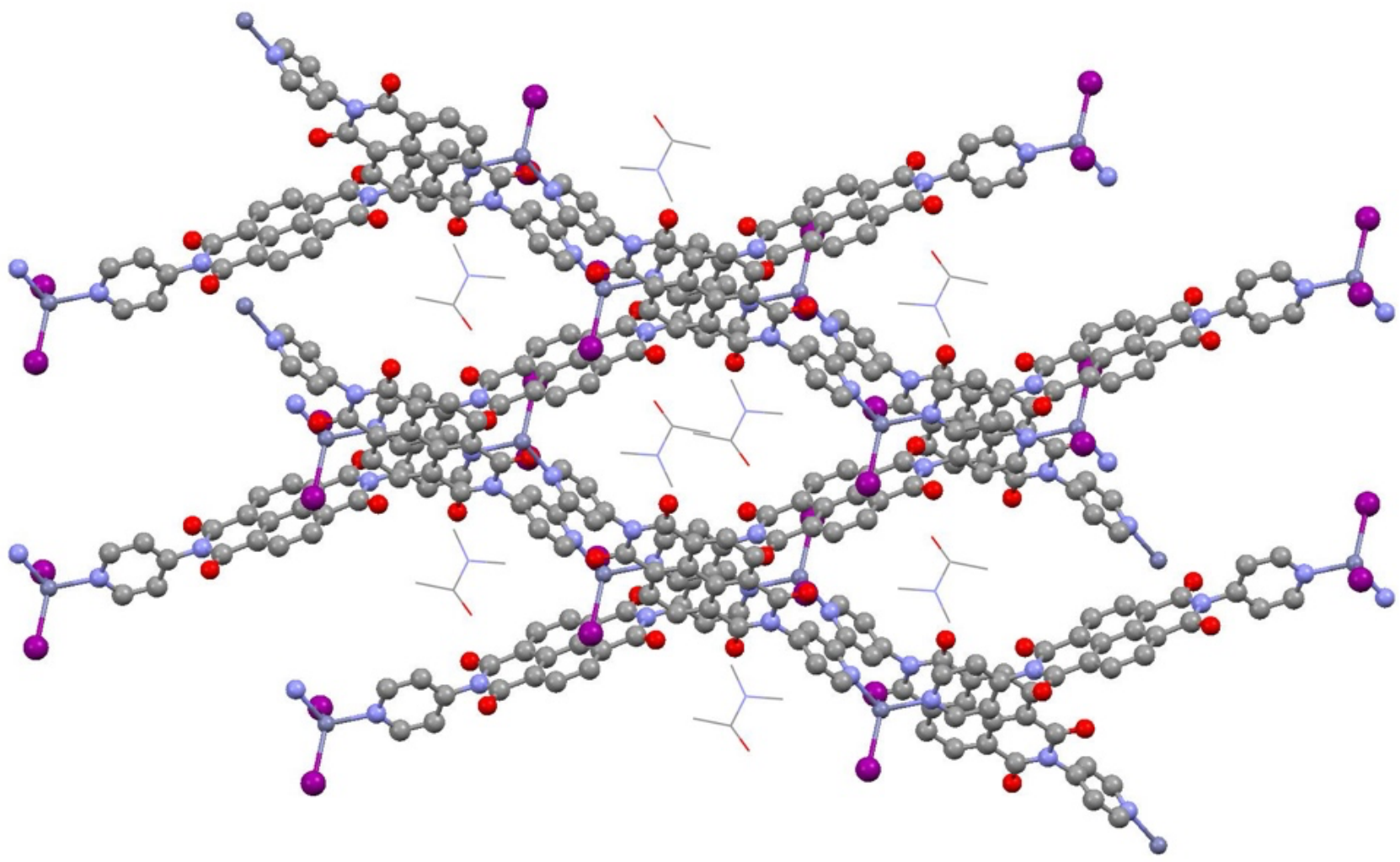

***Figure S6**. View along the c-axis of $[Zn(D4PNDI)I_2]_n$ showing DMAc molecules (wireframe molecules) within pores between CP chains. (C – grey; N – light blue; O – red; I – purple).*

Table S1. Bond lengths and angles for all crystal structures reported.

$[Zn(D4PNDI)Cl_2]_n$

Bond Lengths / Å

Zn1	Cl1^1	2.2158(6)	C7	C6	1.485(3)
Zn1	Cl1	2.2158(6)	C7	C11	1.376(3)
Zn1	N1	2.0671(19)	C9	C12	1.381(3)
Zn1	N1^1	2.0672(19)	C9	C10	1.483(3)
O2	C10	1.213(3)	C12	C11^2	1.402(3)
O1	C6	1.216(3)	C3	C4	1.385(3)
N2	C6	1.400(3)	C3	C2	1.373(3)
N2	C3	1.439(3)	C5	C4	1.382(3)
N2	C10	1.407(3)	C2	C1	1.380(3)
N1	C5	1.338(3)	O100	C101	1.230(4)
N1	C1	1.353(3)	C101	C100	1.501(5)
C8	C8^2	1.417(4)	C101	N100	1.338(4)
C8	C7	1.412(3)	N100	C102	1.467(4)
C8	C9	1.416(3)	N100	C103	1.463(5)

Bond Angles / ˚

Cl1^1	Zn1	Cl1	124.53(4)	C8	C9	C10	119.5(2)
N1	Zn1	Cl1	103.67(6)	C12	C9	C8	120.5(2)
N1^1	Zn1	Cl1	113.26(6)	C12	C9	C10	120.0(2)
N1^1	Zn1	Cl1^1	103.67(6)	C9	C12	C11^2	120.5(2)
N1	Zn1	Cl1^1	113.26(6)	C4	C3	N2	119.0(2)
N1	Zn1	N1^1	94.59(11)	C2	C3	N2	120.80(19)
C6	N2	C3	117.16(18)	C2	C3	C4	120.2(2)
C6	N2	C10	126.05(18)	C7	C11	C12^2	120.2(2)
C10	N2	C3	116.67(18)	O2	C10	N2	120.6(2)

C5	N1	Zn1	118.79(15)	O2	C10	C9	122.9(2)
C5	N1	C1	117.9(2)	N2	C10	C9	116.50(19)
C1	N1	Zn1	121.02(16)	N1	C5	C4	123.0(2)
C7	C8	C8[2]	119.6(2)	C5	C4	C3	117.9(2)
C7	C8	C9	121.66(19)	C3	C2	C1	118.3(2)
C9	C8	C8[2]	118.7(3)	N1	C1	C2	122.6(2)
C8	C7	C6	120.1(2)	O100	C101	C100	118.1(7)
C11	C7	C8	120.5(2)	O100	C101	N100	122.6(5)
C11	C7	C6	119.4(2)	N100	C101	C100	119.3(6)
O1	C6	N2	120.8(2)	C101	N100	C102	123.2(8)
O1	C6	C7	123.1(2)	C101	N100	C103	120.5(6)
N2	C6	C7	116.15(19)	C103	N100	C102	116.2(10)

$[Zn(D4PNDI)Br_2]_n$

Bond Lengths / Å

Br1	Zn1	2.3487(3)	C7	C6	1.486(3)
Zn1	N1[1]	2.066(2)	C7	C11	1.382(4)
Zn1	N1	2.066(2)	C11	C12[2]	1.401(4)
O2	C10	1.208(3)	C9	C12	1.380(4)
O1	C6	1.215(3)	C3	C4	1.382(4)
N2	C10	1.411(3)	C3	C2	1.381(4)
N2	C6	1.400(3)	C4	C5	1.385(4)
N2	C3	1.440(3)	C1	C2	1.386(4)
N1	C1	1.347(3)	O100	C103	1.228(4)
N1	C5	1.341(3)	C103	C100	1.500(5)
C10	C9	1.485(3)	C103	N100	1.332(4)
C8	C8[2]	1.417(5)	N100	C101	1.461(4)
C8	C7	1.413(4)	N100	C102	1.463(5)
C8	C9	1.417(3)			

Bond Angles / °

Br1	Zn1	Br1[1]	123.66(2)	O1	C6	N2	120.7(2)
N1	Zn1	Br1	104.46(6)	O1	C6	C7	123.2(2)
N1	Zn1	Br1[1]	112.58(6)	N2	C6	C7	116.1(2)
N1[1]	Zn1	Br1[1]	104.46(6)	C7	C11	C12[2]	120.2(2)
N1[1]	Zn1	Br1	112.58(6)	C8	C9	C10	119.8(2)
N1[1]	Zn1	N1	95.69(13)	C12	C9	C10	120.1(2)
C10	N2	C3	116.3(2)	C12	C9	C8	120.2(2)
C6	N2	C10	126.0(2)	C9	C12	C11[2]	120.8(2)
C6	N2	C3	117.6(2)	C4	C3	N2	119.3(2)
C1	N1	Zn1	121.63(18)	C2	C3	N2	120.4(2)
C5	N1	Zn1	118.32(18)	C2	C3	C4	120.3(2)
C5	N1	C1	118.1(2)	C3	C4	C5	118.1(2)
O2	C10	N2	120.9(2)	N1	C1	C2	122.9(3)
O2	C10	C9	122.7(2)	C3	C2	C1	117.7(2)
N2	C10	C9	116.4(2)	N1	C5	C4	122.6(2)

C7	C8	C8^{2}	119.7(3)	O100	C103	C100	118.2(8)
C7	C8	C9	121.4(2)	O100	C103	N100	122.3(5)
C9	C8	C8^{2}	119.0(3)	N100	C103	C100	119.5(6)
C8	C7	C6	120.4(2)	C103	N100	C101	125.9(8)
C11	C7	C8	120.2(2)	C103	N100	C102	121.0(6)
C11	C7	C6	119.4(2)	C101	N100	C102	113.0(10)

[Zn(D4PNDI)Cl$_{0.4}$Br$_{1.6}$]$_n$

Bond Lengths / Å

Zn1	Br1	2.3124(12)	C8	C7	1.414(5)
Zn1	Br1^{1}	2.3124(12)	C10	C9	1.482(5)
Zn1	N1^{1}	2.070(3)	C11	C12^{2}	1.402(5)
Zn1	N1	2.070(3)	C11	C7	1.381(5)
Zn1	Cl1	2.178(14)	C6	C7	1.484(5)
Zn1	Cl1^{1}	2.178(14)	C12	C9	1.379(5)
O2	C10	1.214(4)	C3	C4	1.387(5)
O1	C6	1.213(4)	C3	C2	1.377(5)
N2	C10	1.407(5)	C4	C5	1.384(5)
N2	C6	1.410(5)	C2	C1	1.385(5)
N2	C3	1.434(4)	O100	C101	1.228(4)
N1	C1	1.338(5)	C101	C100	1.499(5)
N1	C5	1.344(5)	C101	N100	1.326(4)
C8	C8^{2}	1.408(7)	N100	C102	1.459(5)
C8	C9	1.419(5)	N100	C103	1.461(5)

Bond Angles / °

Br1^{1}	Zn1	Br1	125.39(5)	O1	C6	N2	120.4(3)
N1^{1}	Zn1	Br1	112.21(10)	O1	C6	C7	123.0(3)
N1^{1}	Zn1	Br1^{1}	103.91(9)	N2	C6	C7	116.6(3)
N1	Zn1	Br1^{1}	112.21(10)	C9	C12	C11^{2}	120.2(3)
N1	Zn1	Br1	103.91(9)	C8	C9	C10	120.4(3)
N1^{1}	Zn1	N1	95.23(17)	C12	C9	C8	120.1(3)
N1	Zn1	Cl1	107.7(4)	C12	C9	C10	119.5(3)
N1	Zn1	Cl1^{1}	117.5(4)	C8	C7	C6	119.9(3)
N1^{1}	Zn1	Cl1^{1}	107.7(4)	C11	C7	C8	120.2(3)
N1^{1}	Zn1	Cl1	117.5(4)	C11	C7	C6	119.8(3)
Cl1^{1}	Zn1	Cl1	110.7(6)	C4	C3	N2	118.9(3)
C10	N2	C6	125.7(3)	C2	C3	N2	121.1(3)
C10	N2	C3	117.6(3)	C2	C3	C4	120.1(3)
C6	N2	C3	116.6(3)	C5	C4	C3	117.8(3)
C1	N1	Zn1	121.4(3)	C3	C2	C1	118.1(3)
C1	N1	C5	118.1(3)	N1	C1	C2	122.8(4)
C5	N1	Zn1	118.4(2)	N1	C5	C4	122.8(3)
C8^{2}	C8	C9	119.5(4)	O100	C101	C100	116.7(8)
C8^{2}	C8	C7	119.3(4)	O100	C101	N100	122.7(6)

C7	C8	C9	121.2(3)	N100	C101	C100	120.6(7)
O2	C10	N2	120.6(3)	C101	N100	C102	127.0(8)
O2	C10	C9	123.2(3)	C101	N100	C103	121.8(6)
N2	C10	C9	116.2(3)	C102	N100	C103	111.1(10)
C7	C11	C12^2	120.6(3)				

[Zn(D4PNDI)I_2]$_n$

Bond Lengths / Å

I2	Zn1	2.5792(8)	C102	C103	1.383(7)
I1	Zn1	2.5360(8)	C102	C101	1.389(7)
Zn1	N100	2.048(4)	C12	C12^2	1.407(9)
Zn1	N1	2.072(4)	C12	C11	1.408(7)
O101	C109	1.221(6)	C12	C8^2	1.414(7)
O1	C6	1.223(6)	C11	C10	1.380(7)
O100	C105	1.210(6)	C10	C9	1.406(7)
O2	C7	1.222(7)	C100	C101	1.391(7)
N101	C109	1.403(7)	C8	C9	1.384(7)
N101	C102	1.434(6)	C8	C7	1.473(7)
N101	C105	1.394(6)	C106	C110	1.371(7)
N100	C100	1.333(7)	C106	C105	1.479(7)
N100	C104	1.350(6)	C103	C104	1.377(7)
N2	C6	1.395(7)	C110	C111^1	1.407(7)
N2	C3	1.442(6)	C3	C4	1.379(8)
N2	C7	1.402(7)	C3	C2	1.377(8)
N1	C1	1.334(8)	C1	C2	1.385(7)
N1	C5	1.343(8)	C5	C4	1.384(7)
C109	C108	1.480(7)	O200	C201	1.228(5)
C107	C107^1	1.413(9)	C201	C200	1.508(5)
C107	C108	1.407(7)	C201	N200	1.326(5)
C107	C106	1.420(7)	N200	C202	1.460(5)
C108	C111	1.371(7)	N200	C203	1.458(5)
C6	C11^2	1.478(7)			

Bond Angles / °

I1	Zn1	I2	122.64(3)	C12	C11	C6^2	119.9(4)
N100	Zn1	I2	103.01(12)	C10	C11	C6^2	119.5(5)
N100	Zn1	I1	107.66(12)	C10	C11	C12	120.5(4)
N100	Zn1	N1	109.06(18)	C11	C10	C9	120.0(5)
N1	Zn1	I2	105.90(14)	N100	C100	C101	123.1(5)
N1	Zn1	I1	108.00(14)	C12^2	C8	C7	120.1(5)
C109	N101	C102	117.0(4)	C9	C8	C12^2	120.2(5)
C105	N101	C109	125.2(4)	C9	C8	C7	119.7(5)
C105	N101	C102	117.6(4)	C8	C9	C10	120.4(5)

C100	N100	Zn1	124.3(3)	C107	C106	C105	119.2(4)
C100	N100	C104	118.1(4)	C110	C106	C107	120.6(5)
C104	N100	Zn1	117.1(3)	C110	C106	C105	120.1(5)
C6	N2	C3	117.7(4)	C104	C103	C102	117.9(5)
C6	N2	C7	125.4(4)	N100	C104	C103	123.0(5)
C7	N2	C3	116.6(4)	C106	C110	C111[1]	119.9(5)
C1	N1	Zn1	121.2(4)	C102	C101	C100	117.5(5)
C1	N1	C5	117.7(5)	C4	C3	N2	119.9(5)
C5	N1	Zn1	120.2(4)	C2	C3	N2	120.1(5)
O101	C109	N101	120.0(4)	C2	C3	C4	120.1(5)
O101	C109	C108	123.1(5)	C108	C111	C110[1]	120.7(5)
N101	C109	C108	116.9(4)	O2	C7	N2	120.1(5)
C107[1]	C107	C106	118.9(6)	O2	C7	C8	123.2(5)
C108	C107	C107[1]	119.5(6)	N2	C7	C8	116.6(4)
C108	C107	C106	121.6(4)	O100	C105	N101	119.2(4)
C107	C108	C109	119.7(4)	O100	C105	C106	123.5(5)
C111	C108	C109	119.9(4)	N101	C105	C106	117.3(4)
C111	C108	C107	120.4(4)	N1	C1	C2	122.9(5)
O1	C6	N2	120.0(4)	N1	C5	C4	123.3(5)
O1	C6	C11[2]	123.3(5)	C3	C4	C5	117.6(5)
N2	C6	C11[2]	116.7(4)	C3	C2	C1	118.2(5)
C103	C102	N101	120.2(4)	O200	C201	C200	135.9(13)
C103	C102	C101	120.2(5)	O200	C201	N200	117.9(11)
C101	C102	N101	119.5(4)	N200	C201	C200	106.2(12)
C12[2]	C12	C11	119.7(6)	C201	N200	C202	130.0(14)
C12[2]	C12	C8[2]	119.2(6)	C201	N200	C203	111.2(12)
C11	C12	C8[2]	121.1(4)	C203	N200	C202	118.4(15)

Table S2. Crystallographic information for data collections of coordination polymers $[Zn(D4PNDI)Cl_2]_n$, $[Zn(D4PNDI)Br_2]_n$, $[Zn(D4PNDI)Cl_{0.4}Br_{1.6}]_n$ and $[Zn(D4PNDI)I_2]_n$

Crystal Structure	$[Zn(D4PNDI)Cl_2]_n$	$[Zn(D4PNDI)Br_2]_n$	$[Zn(D4PNDI)Cl_{0.4}Br_{1.6}]_n$	$[Zn(D4PNDI)I_2]_n$
Empirical Formula	$C_{28}H_{21}Cl_2N_5O_5Zn$	$C_{28}H_{21}Br_2N_5O_5Zn$	$C_{28}H_{21}Br_{1.6}Cl_{0.4}N_5O_5Zn$	$C_{30}H_{29}I_2N_6O_5Zn$
Formula Weight	643.77	732.69	714.9	872.76
Temperature	100	100	100	100
Crystal System	monoclinic	monoclinic	monoclinic	triclinic
Space Group	P2/c	P2/c	P2/c	P-1
a/Å	7.46340(10)	7.50620(10)	7.48780(10)	11.9910(2)
b/Å	12.2391(2)	12.3590(2)	12.3164(2)	12.1137(2)
c/Å	14.8451(2)	15.0908(3)	14.9819(2)	14.5675(2)
α/°	90	90	90	94.1057(13)
β/°	94.633(2)	94.471(2)	94.4600(10)	111.4036(16)
γ/°	90	90	90	112.4535(16)
Volume/Å^3	1351.60(3)	1395.70(4)	1377.49(3)	1765.14(6)
Z	2	2	2	2
ρ_{calc}g/cm^3	1.582	1.743	1.724	1.642
μ/mm^{-1}	3.505	4.988	4.731	15.066
Crystal size/mm^3	0.15 × 0.04 × 0.02	0.15 × 0.03 × 0.03	0.11 × 0.04 × 0.02	0.29 × 0.06 × 0.06
Radiation	Cu Kα (λ = 1.54184)			
2Θ range for data collection/°	7.222 to 159.598	7.152 to 158.692	7.178 to 158.12	8.146 to 155.188
Reflections collected	14218	14658	25788	50252
Independent reflections	2867 [R_{int} = 0.0542, R_{sigma} = 0.0397]	2953 [R_{int} = 0.0447, R_{sigma} = 0.0324]	2964 [R_{int} = 0.0492, R_{sigma} = 0.0233]	7054 [R_{int} = 0.0542, R_{sigma} = 0.0269]
Data/restraints/parameters	2867/15/216	2953/15/216	2964/15/219	7054/45/374
Goodness-of-fit on F^2	1.055	1.095	1.064	1.029
Final R indexes [I>=2σ (I)]	R_1 = 0.0362, wR_2 = 0.0900	R_1 = 0.0326, wR_2 = 0.0871	R_1 = 0.0488, wR_2 = 0.1358	R_1 = 0.0499, wR_2 = 0.1344
CCDC Deposition Number	2560042	2560041	2560072	2579247

Interestingly, the unit cell parameters of the mixed-halide crystal are intermediate to those of the fully chloride and bromide structures, this is also observed in the PXRD studies.

Diffuse reflectance UV-Vis spectroscopy

The photoexcitation of the $ZnCl_2$ and $ZnBr_2$ coordination polymers were probed by irradiating a powdered sample over various time periods and collecting the UV-Vis spectrum between 250 and 900 nm. The decay kinetics of the coordination polymers were probed by irradiating samples for 120 s, then collecting the UV-Vis spectra over the course of 2000 s at 40 s intervals.

The effect of Br percentage in the overall composition of the material was investigated by irradiating samples of varying composition (as confirmed by EDX) for 300 s (to ensure maximum radical population) and collecting the UV-Vis spectra between 250 and 900 nm.

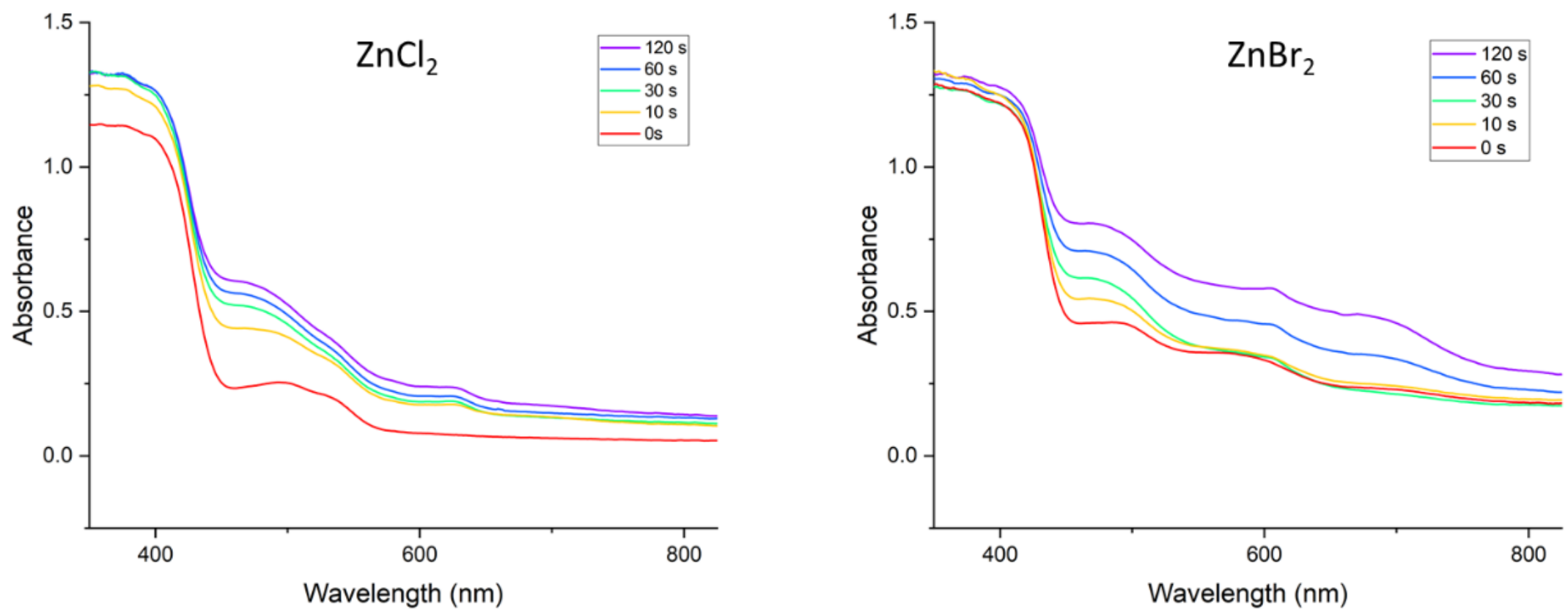

***Figure S7**. UV-Vis spectra on the photoexcited $ZnCl_2$ and $ZnBr_2$ coordination polymers at various irradiation times.*

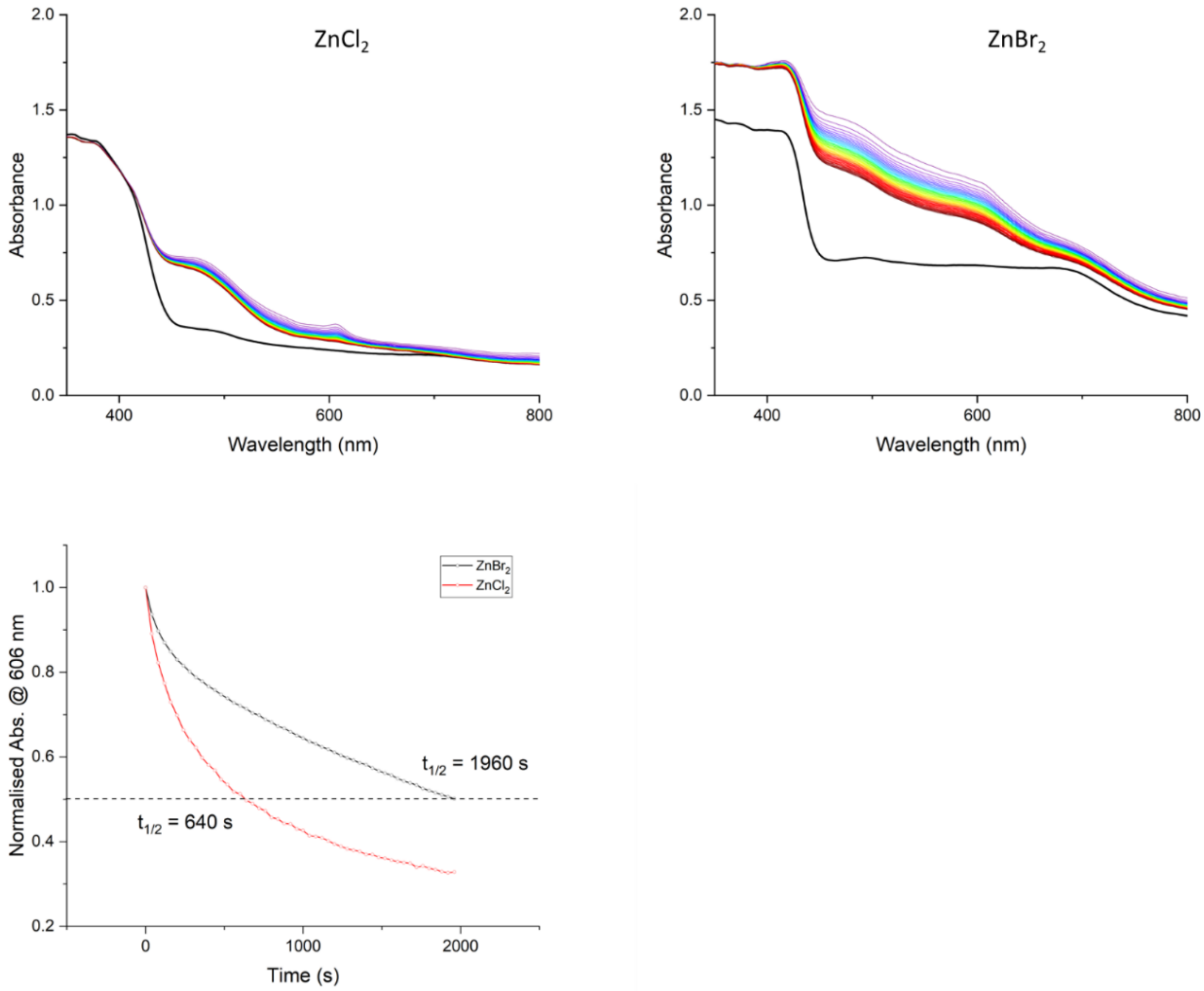

***Figure S8**. Reversal of photochromism in the dark, measured between 0 and 2000s. In particular the decay of the NDI radical peak at 606 nm is plotted against time to demonstrate the stability of the radical within the respective structures.*

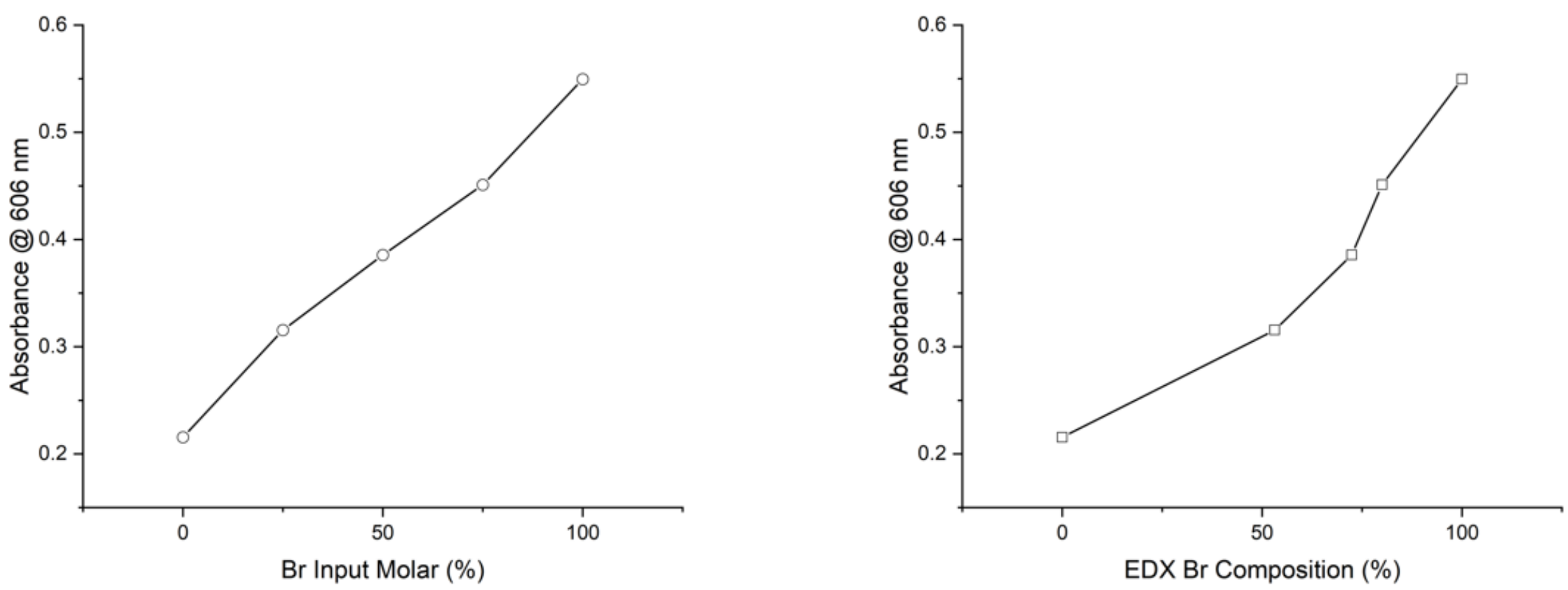

***Figure S9**. The relationship between Br input molar % and measured Br composition by EDX on the absorbance of the peak at 606 nm in multivariate coordination polymers after photoirradiation by 365 nm for 300 seconds.*

All samples were irradiated by a 365 nm UV lamp and spectra were collected on a Shimadzu UV-3600i spectrometer fitted with an integrating sphere for diffuse reflectance measurements. All measurements were referenced to a $BaSO_4$ standard.

Energy Dispersive X-ray Spectroscopy (EDX)

Samples for EDX were mounted upon a carbon backing and data was collected on a Zeiss Evo 15 electron microscope fitted with a Bruker Quantax Esprit-X Flash 6160 EDX detector. EDX spectra were collected at an accelerating voltage of 30 kV and probe current of 3 nA over the course of 60 seconds at a working distance of 9 mm. The probe current was set to 1 nA for EDX mapping in order to attain improved special resolution. All EDX maps were collected over 10 minutes.

EDX quantification was performed using a P/B-ZAF quantification method within the Bruker Esprit software package to account for the atomic number, absorption and fluorescence effects within the sample which would be expected to be larger for bromine compared to chlorine. The EDX measurements are accurate within 2%.

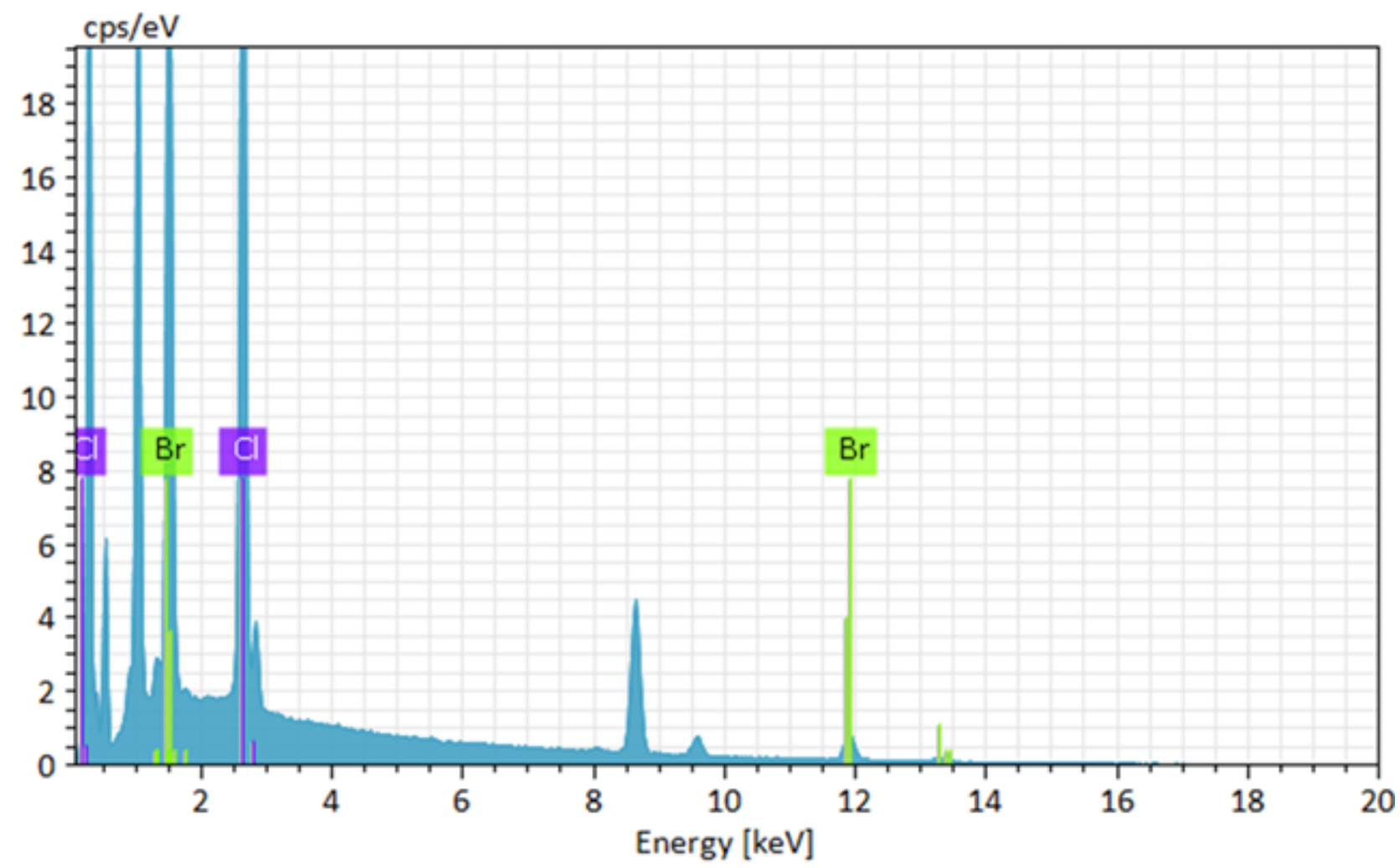

Figure S10. *EDX Spectrum of* $[Zn(D4PNDI)Cl_{0.94}Br_{1.06}]_n$

To validate the quantification methodology, control experiments were performed on the end-member compositions $[Zn(D4PNDI)Cl_2]_n$ and $[Zn(D4PNDI)Br_2]_n$. The EDX results showed no detectable atomic concentrations of the complementary halides in these samples confirming the reliability of the quantification method and excluding contamination as a source of error in the mixed halide composition measurements.

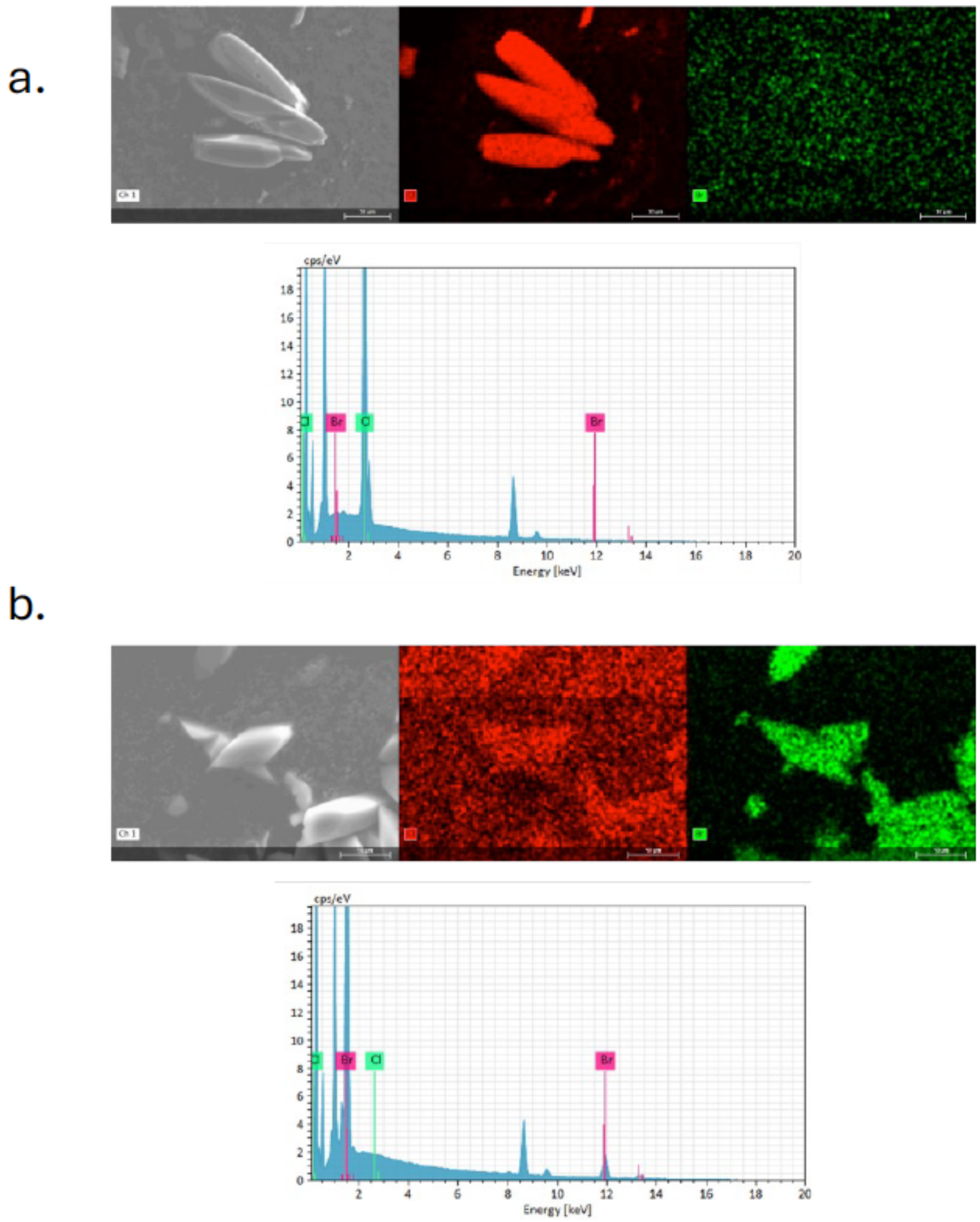

Figure S11. *EDX control experiments verify reliability of quantification model for halides, a) [Zn(D4PNDI)Cl$_2$]$_n$ and b) [Zn(D4PNDI)Br$_2$]$_n$. Notably the spectra and maps show no signal for the halide which was not present in the synthesis of the crystals.*

Computational Modelling

Density functional theory (DFT) calculations were performed using ORCA 6.1.1 on a home PC (AMD Ryzen 5 3600 6 core CPU, 16 GB DDR4 RAM @ 1600 MHz, AMD Radeon 5600 XT GPU). All .ins, .out and .xyz files used for the calculations are made available to the reader. Molecular orbitals were plotted onto the molecular cluster models using Vesta.

Table S3. 11 highest energy molecular orbitals calculated for $ZnCl_2$, $ZnBr_2$ and hypothetical ZnI_2 clusters. Note that MO numbering is not directly comparable for the ZnI_2 calculation as while $ZnCl_2$ and $ZnBr_2$ were treated as all-electron, ZnI2 has I with a Def2-ECP (28 electron effective core potential).

**MO**	**MO Description**	**$ZnCl_2$ Cluster — MO Energies (B3LYP/def2-SVP)**			**$ZnBr_2$ Cluster — MO Energies (B3LYP/def2-SVP)**			**ZnI_2 Cluster — MO Energies (B3LYP/def2-SVP)**		
		**MO #**	**E (Eh)**	**E (eV)**	**MO #**	**E (Eh)**	**E (eV)**	**MO #**	**E (Eh)**	**E (eV)**
HOMO - 9		172	-0.2702	-7.3526	190	-0.2681	-7.2948	180	-0.2679	-7.2893
HOMO - 8		173	-0.2674	-7.2762	191	-0.2662	-7.2434	181	-0.2651	-7.2145
HOMO - 7		174	-0.2658	-7.2316	192	-0.2647	-7.2028	182	-0.2575	-7.008
HOMO - 6		175	-0.2562	-6.9723	193	-0.2562	-6.9715	183	-0.2553	-6.9464
HOMO - 5		176	-0.2547	-6.932	194	-0.255	-6.9378	184	-0.2493	-6.7832
HOMO - 4		177	-0.2511	-6.834	195	-0.2503	-6.8102	185	-0.2431	-6.6162
HOMO - 3		178	-0.2504	-6.815	196	-0.242	-6.5843	186	-0.2244	-6.1052
**HOMO - 2**	***Strong halide LP character***	**179**	**-0.2458**	**-6.6892**	**197**	**-0.2382**	**-6.4827**	**187**	**-0.222**	**-6.0397**
**HOMO - 1**	***Strong halide LP character***	**180**	**-0.237**	**-6.4488**	**198**	**-0.2299**	**-6.2552**	**188**	**-0.21**	**-5.7148**
**HOMO**	***Strong halide LP character***	**181**	**-0.2333**	**-6.3493**	**199**	**-0.226**	**-6.1485**	**189**	**-0.2019**	**-5.4934**
**LUMO**	***NDI LUMO***	**182**	**-0.1223**	**-3.3266**	**200**	**-0.1222**	**-3.3264**	**190**	**-0.1213**	**-3.3006**
**HOMO–LUMO Gap**			**0.1110**	**3.0227**		**0.1038**	**2.8221**		**0.0806**	**2.1928**

Powder X-ray Diffraction (PXRD) Studies

PXRD diffractograms were collected on an empyrean PANalytical diffractometer using a Cu X-ray source between 2° and 40° 2θ. The diffractograms confirm that the coordination polymers of the various halide compositions are isostructural. Comparison of the diffractograms with simulated powder patterns confirm phase purity.

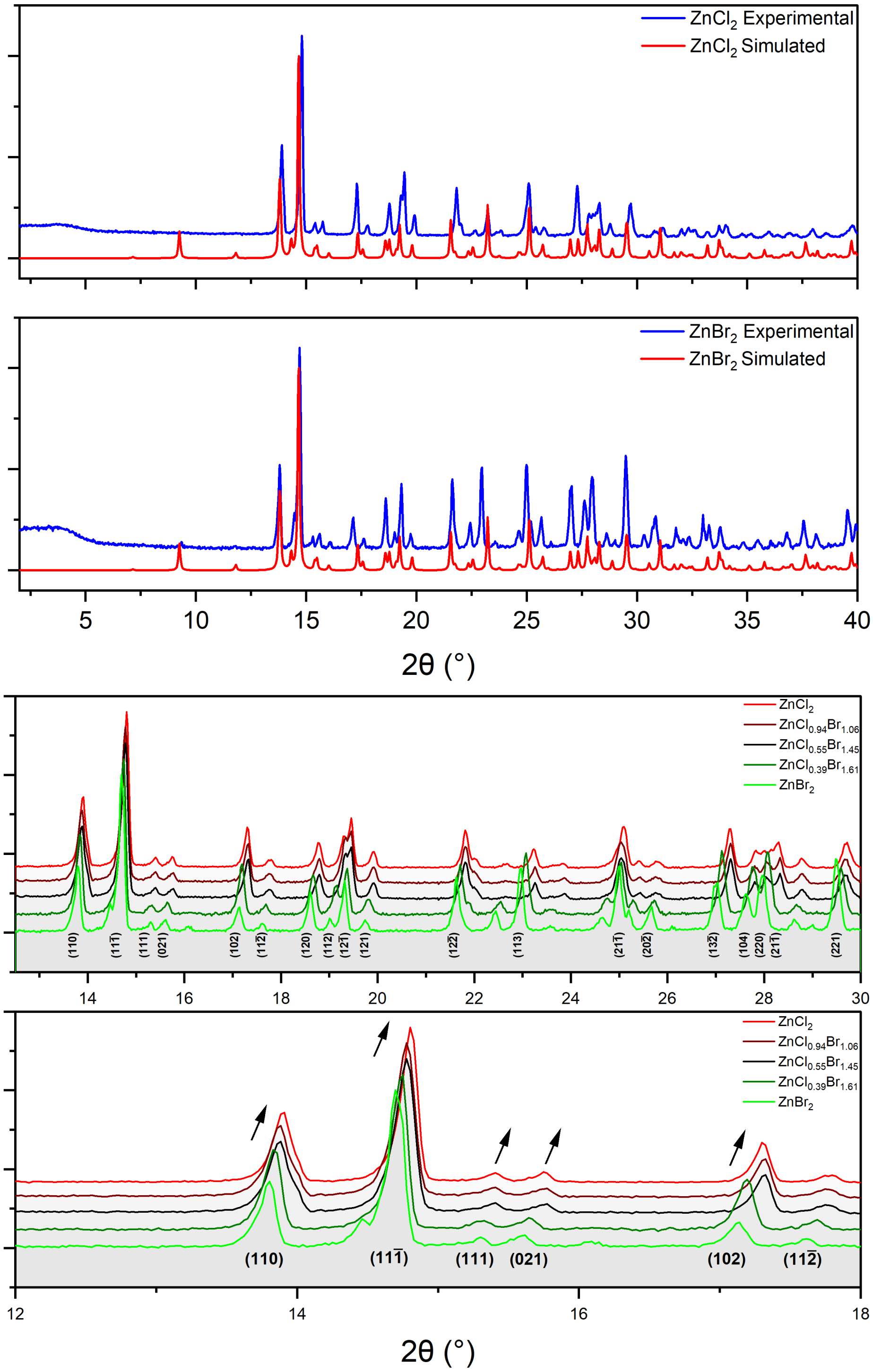

***Figure S12**. PXRD diffractograms comparing $ZnCl_2$ and $ZnBr_2$ to multivariate examples shows isostructural nature. Simulated diffractograms compared to experimental data confirm phase purity and isostructurality.*

As observed in the single crystal studies with *$[Zn(D4PNDI)Cl_{0.4}Br_{1.6}]_n$* having intermediate unit cell parameters to the $ZnCl_2$ and $ZnBr_2$ single crystal structures, we also observe a gradual contraction of unit cell parameters in PXRD studies as chloride composition increases as evidenced by a shifting of reflections towards higher values of 2θ.

Thermogravimetric Analysis (TGA)

TGA was conducted on a Perkin-Elmer TGA8000 instrument. Samples were heated from 50 °C to 800 °C at a rate of 5 °C per minute. An initial mass loss is observed between 13 and 17% depending on CP composition beginning at 180 °C and ending at 230 °C. This mass loss is consistent with approximately 2 DMAc molecule per unit cell, which is consistent with the x-ray structures. A second larger mass loss is observed beginning at 370 °C to 700 °C which is attributed to thermal degradation of the coordination polymer.

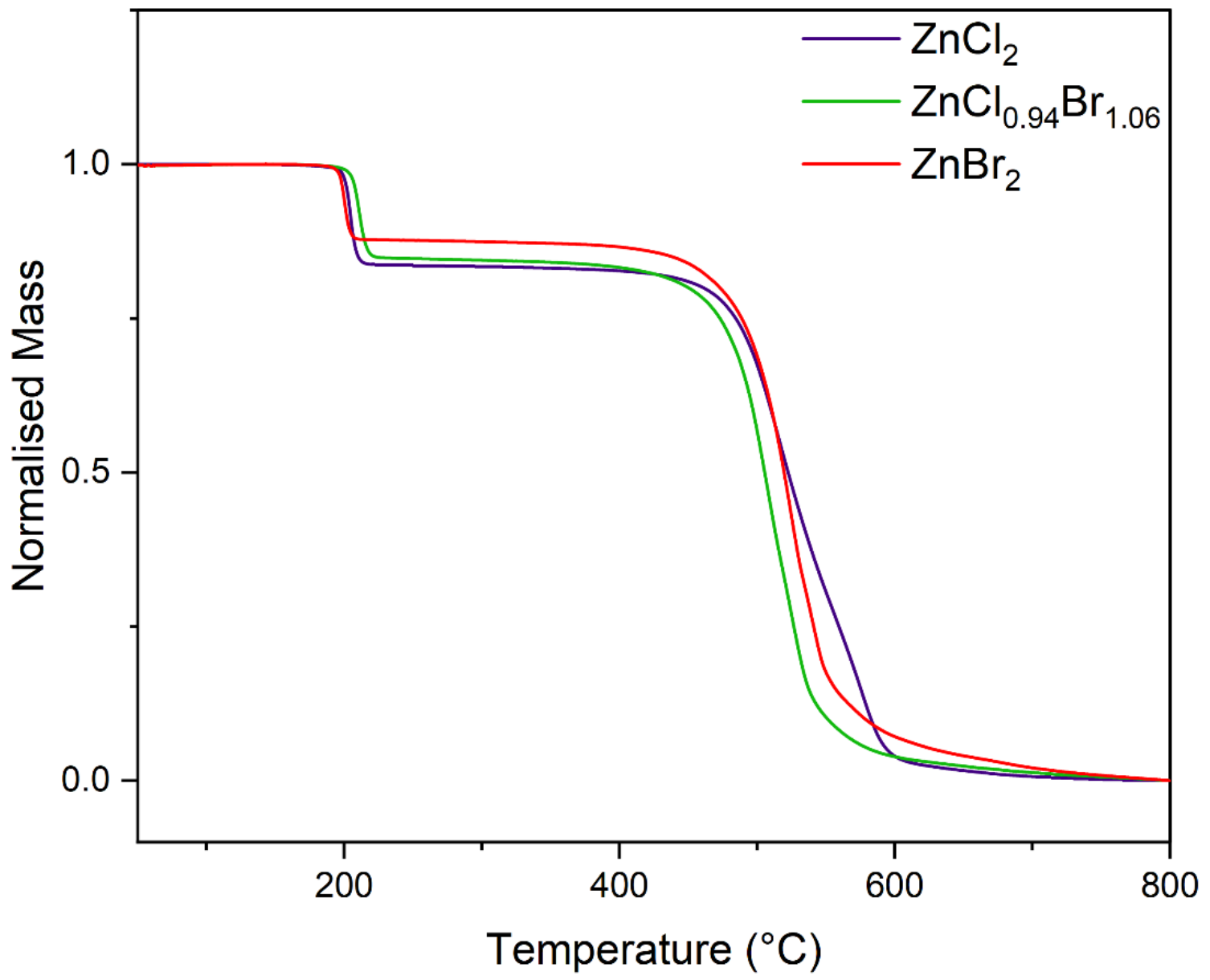

***Figure S13**. TGA plots for $[Zn(D4PNDI)Cl_2]_n$, $[Zn(D4PNDI)Br_2]_n$ and $[Zn(D4PNDI)Cl_{0.4}Br_{1.6}]_n$.*

Solid-state FTIR spectroscopy

ATR-FTIR spectra were recorded on powdered samples of the coordination polymers using a PerkinElmer spectrum two spectrometer. Spectra were collected between 400 and 4000 cm^{-1} however, figures were produced between 400 and 2000 cm^{-1} to aid in clarity of the fingerprint region.

Analysis of the respective ground state spectra shows that the materials are functionally identical, suggesting that the identity of the halide has an undetectable effect on the NDI in the ground state using this technique. Characteristic stretches of the NDI C=O imide groups can be observed clearly at 1720 cm^{-1} however, attempts to collect spectra upon irradiation of the sample by a 365 nm UV lamp yielded essentially identical spectra which can be attributed to the relatively weak photochromic response, compared to similar NDI systems we have investigated in other studies.

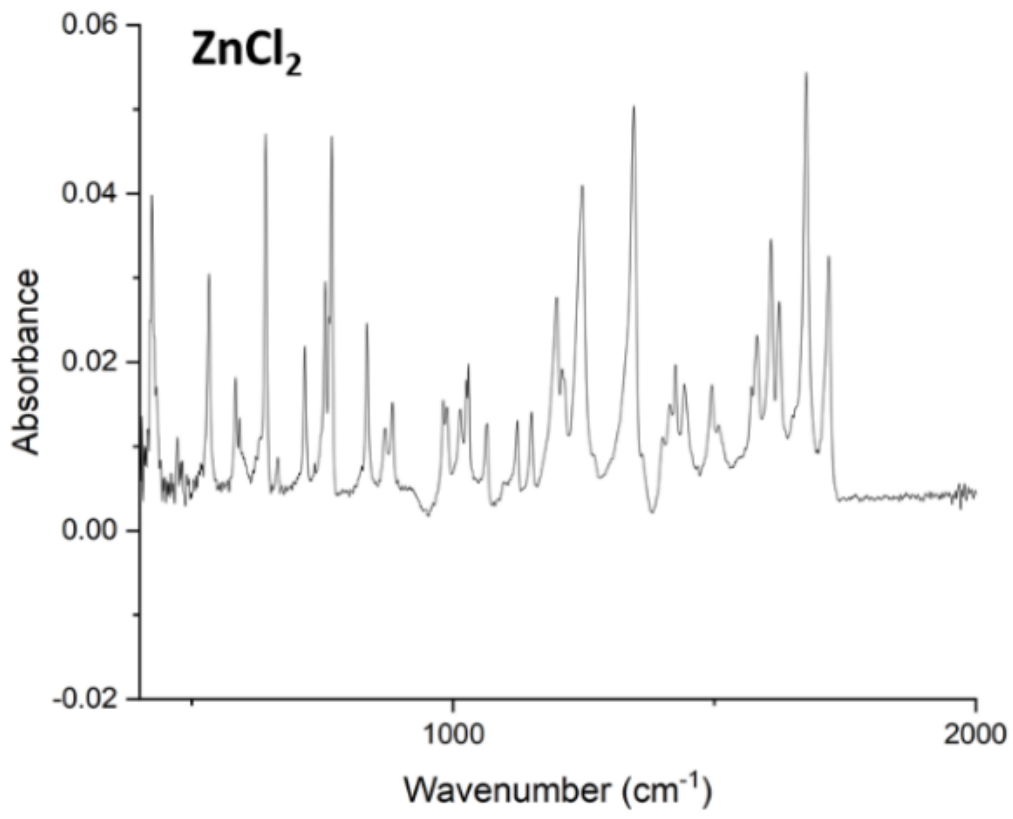

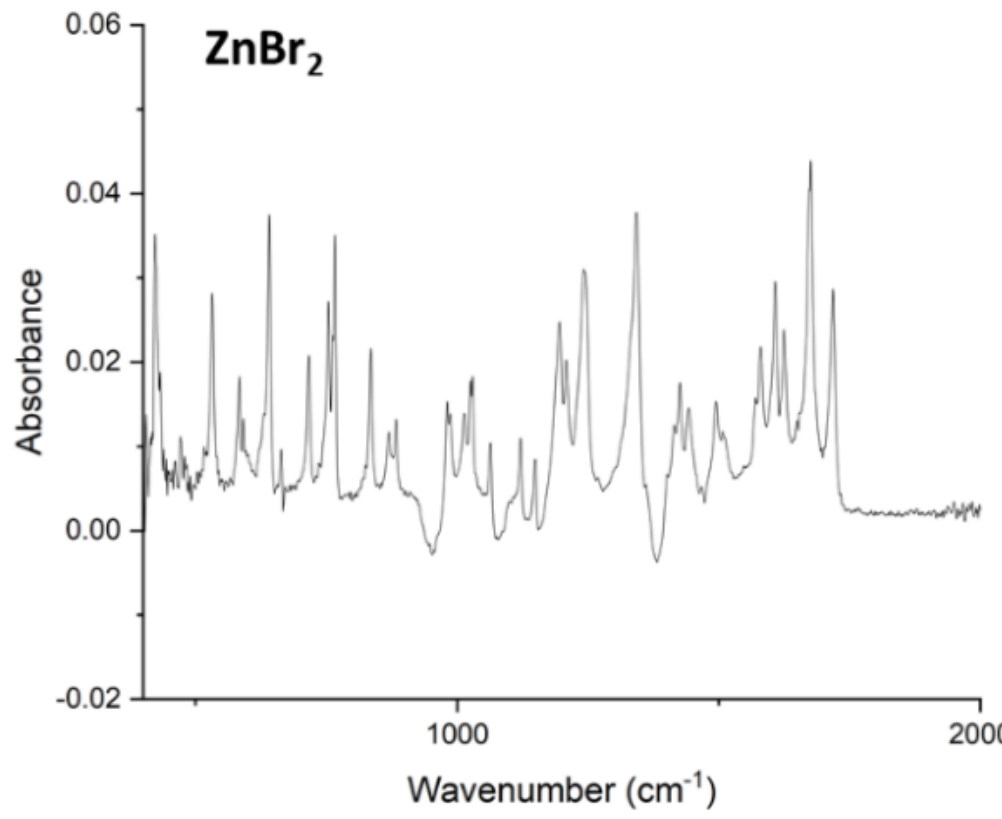

***Figure S14**. ATR-FTIR spectra of $ZnCl_2$ and $ZnBr_2$ coordination polymer powdered crystals.*

References

S1 A. Ishaq, J.O. Ogar, M.M. Mahmud D.E. Schier, S.P. Argent, N.R. Champness, 2026, *submitted*.

S2 CrysAlisPro, Rigaku Oxford Diffraction, 2020 & 2021.

S3 D. R. Allan et al., *Crystals* 2017, *7*(11), 336.

S4 J. Beilsten-Edmands et al., *Acta Cryst*. 2020, D76, 385-399; P. Evans, *Acta Cryst*. 2006, D62, 72-82; P.R. Evans and G.N. Murshudov, *Acta Cryst*. 2013, D69, 1204-1214; M. D. Winn et al., *Acta Cryst*. 2011, D67, 235-242; G. Winter, *J. Appl. Cryst*. 2010, 43, 186-190; G. Winter et al., *Acta Cryst*. 2018, D74, 85-97.

S5 O. V. Dolomanov, L. J. Bourhis, R. J. Gildea, J. A. K. Howard, H. Puschmann, *J. Appl. Crystallogr.* 2009, *42*, 339-341.

S6 G. M. Sheldrick, *Acta Cryst.* 2015, *A71*, 3-8.

S7 G. M. Sheldrick, *Acta Cryst.* 2015, *C71*, 3-8.
